# Smart insect trap: insect identification and classification using iNaturalist and ChatGPT

**DOI:** 10.1098/rsos.251212

**Published:** 2026-10-28

**Authors:** Yuhan Wu, Anthony Nicolaides, Anthony Coffin-Schmitt, Sunghwan Jung

**Affiliations:** ^1^ Department of Electrical and Computer Engineering, Cornell University, Ithaca, NY, USA; ^2^ Department of Biological and Environmental Engineering, Cornell University, Ithaca, NY, USA; ^3^ Department of Computer Science, Cornell University, Ithaca, NY, USA

**Keywords:** insect identification, machine learning, computer vision, object detection, data augmentation, iNaturalist, ChatGPT, pest control, agricultural engineering, agentic AI

## Abstract

Insect identification is crucial for sustainable agriculture and pest control, as it helps determine the pest type, reduces human labour, and decreases reliance on pesticides. However, there are a number of challenges associated with AI-based insect identification and classification, such as the lack of reliable data sources, imbalanced datasets and difficulty of annotation. Our paper offers a practical solution to these challenges using online platforms. Specifically, we coordinate multiple AI tools in an agentic-AI-style workflow: iNaturalist's computer vision model identifies the insect, ChatGPT determines whether it is a pest and the result drives a low-cost robotic trap. This approach is particularly accessible for non-experts, as it does not require extensive knowledge of machine learning or computer vision, and each component can be upgraded as better AI tools emerge. The system was further validated through field testing using images collected under real agricultural conditions. The results show our method is able to identify and differentiate insect species with sufficient accuracy. Our approach would be more accessible, reliable and efficient than traditional methods and can be used to identify a wider range of insect species.

## 1. Introduction

Insect identification plays a critical role in agriculture and pest management, guiding the selection of appropriate control strategies [1–3]. Traditional insect identification methods typically rely on human expertise and are often time-consuming and prone to errors [4].

Automatic insect detection and identification systems utilizing AI are in the early stages of development and several approaches are being pursued [5]. For instance, mobile devices that interface with cloud platforms to identify agricultural insect pests [6,7], using deep learning and other neural network methods to improve pest detection models [8–10], and insect traps with deep learning models are implemented [11]. Another study also used iNaturalist for insect identification and surveillance but did not focus on pest management [12].

For object detection models for insect identification and classification, cloud-based machine learning platforms like Microsoft Azure's CustomVision.ai offer a cost-effective and accessible solution [13,14]. However, they are extremely data-dependent, require manual annotation and have data limitations, e.g. a maximum image number [15,16]. iNaturalist is a non-profit crowdsourced species identification database [17]. It has acquired a sizable collection of ‘research grade’ image observations and has made CV classification models to identify uploaded observations [18].

iNaturalist's most recent model, v2.21, includes over 101 000 taxa, achieving an accuracy of 90–92% for insect identification with only the image and approximately 95% when geolocation metadata are included [19]. By May 2025, iNaturalist had recorded over 250 million verifiable observations [20]. By utilizing this large and continuously updating database, our device is capable of reliably identifying insects without the need to manually annotate and train an object detection model. This study leverages the iNaturalist database for insect detection and classification and subsequently integrates it into a robotic system for real world deployment. This fusion of computer vision and robotics presents innovative possibilities for pest control in agriculture and ecology. This approach reduces the time and cost associated with building and maintaining insect identification CV models, making it a scalable solution that bridges the gap between advanced AI tools and practical real world application for sustainable pest management in agriculture.

In this paper, we aim to provide a practical approach to overcome common challenges using customized AI algorithms. These challenges involve finding trustworthy data sources, dealing with imbalanced datasets of insect taxonomy and handling the complexities of annotations. Our study is especially helpful for people who are not experts in computer vision (CV) and deep learning. It guides them in making the most of the platform for their needs. This research also contributes to the broader effort in making machine learning more accessible and practical for various domains.

## 2. Material and methods

### 2.1. Raspberry Pi initialization

Operating on the Raspberry Pi OS (Debian, version 12), a main Python script initializes the various components of the device (figure 1A). The main script has a reboot monitor, also called a ‘heartbeat monitor’, that will restart the Raspberry Pi if the system stalls, limiting the necessary hands-on maintenance. The primary components controlled include the Raspberry Pi Camera Module 3, v1.3, LED lights, servo motors and a web driver.

**Figure 1.**
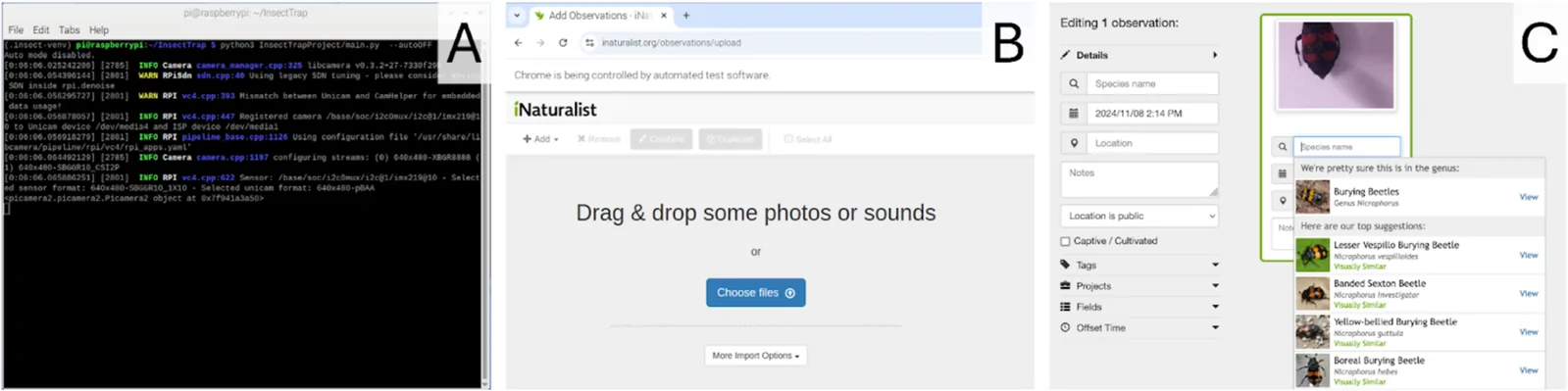
Insect identification pipeline using iNaturalist. In application, the Insect Catcher would run ‘headless’, i.e. without output to a display. However, to visualize the initialization and classification process, the output is displayed. (A) Main Python script initializes and checks the components of the Insect Catcher. (B) After logging into iNaturalist through a web driver, the device is ready to upload the captured images. (C) iNaturalist uses CV model to classify the insect at the lowest taxonomic level with sufficient confidence (see the video in the supplementary material).

The main script interfaces with the camera via the package PiCamera2 to produce a 640 × 480 RGB JPG image. A smaller image size was used compared with the maximum size available on the camera to enable faster processing, yet still provide enough detail for classification. Raspberry Pi Infrared cameras are also available in the same form factor and could be interchanged to enable dim-lighting classification as some insects are primarily nocturnal.

The LEDs, both in the funnel and chamber, are controlled in coordination with the camera or can be set to remain on in low-light settings. Visible light LEDs can be supplemented or replaced with infrared LEDs to facilitate capturing images at night. Ultraviolet (UV) LEDs were added on top for field tests, as some insects are more attracted to UV light.

The servo motors were initialized using the Python library ‘pigpio’, which allows for the control of the General-Purpose Input/Outputs (GPIO) on the Raspberry Pi. The last component initialized was a Python WebDriver, specifically the package Selenium. This web driver accesses the iNaturalist website, logs in with a previously established user account and loads the observation upload web page.

### 2.2. Image identification and pest detection

With the Raspberry Pi components initialized, the camera was set to capture images at 5-second intervals. Once an image is captured and saved to the SD card, the web driver uploads the image to the iNaturalist observation page (figure 1B) and waits for taxa classification by the iNaturalist CV model v2.17. The model returns the most specific taxa-level classification with a confident inference. Ideally, this will be at the species or genus level; however, a family level identification is often sufficient in insect pest management. The device obtains the scientific name, common name (if present) and an iNaturalist taxa identification number. If the model does not have sufficient confidence, no identification is made; however, it offers possible suggestions. Our device only considers the primary identification and logs the suggestions. The metadata of the captured image can also be modified to include geolocation information, which will allow a slight accuracy improvement in the model predictions (figure 1C).

### 2.3. Image classification

Although several online pest tracker lists and reference databases exist for pest classification, they are often incomplete, inconsistent or not directly aligned with iNaturalist's taxonomic nomenclature. Moreover, the classification of an organism as a ‘pest’ depends on a variety of factors, including geographic region, target cop, regulatory status and so on. Given these complexities, a fixed pest dictionary is relatively inflexible and insufficient for broader application scenarios. To address this, we integrated multiple AI-enabled tools into the identification and classification workflow. The system uses a browser-based interface to interact with iNaturalist for visual species identification and OpenAI to provide context-aware pest classification.

Our approach can also be viewed as a multi-tool AI workflow conceptually related to the agentic AI paradigm that has recently gained attention. Rather than relying on a single model, the system sequentially combines browser-based access to iNaturalist, vision-based insect identification and ChatGPT-based pest classification, and the resulting information is used to control the physical device.

Using the insect identification result from the iNaturalist website, the device extracts the most likely scientific name of the taxon. A prompt in the format ‘Is [Insect Scientific Name] a pest or not? Answer me only with YES or NO (if you are uncertain, answer UNCERTAIN) without a comma.’ is sent to ChatGPT. Considering that pest status is highly region-, crop- and time-dependent, we could incorporate additional parameters, including location, crop name, time and other relevant contexts, into the prompt to help refine the classification. The expanded prompt format was: ‘For **Location: [location], time: [time], crop: [crop scientific name], is [Insect Scientific Name] a pest or not? Answer me only with YES or NO (if you are uncertain, answer UNCERTAIN) without comma’. This design is extensible, allowing additional parameters to be incorporated in future work through simple modifications to the model code.

The model is instructed to return ‘YES’, ‘NO’ or ‘UNCERTAIN’, corresponding respectively to the organism being a known pest, not a pest or having pest status that varies by subspecies, region or other contextual factors. We also provide a more detailed definition of the ‘UNCERTAIN’ category; for example, this category may be used in cases where some species within a taxonomic subgroup are pests while others are not, or where regulatory lists such as the U.S. Regulated Plant Pest List may apply to specific jurisdictions but not universally. Based on the returned classification, the insects remain categorized into three classes— ‘YES’, ‘NO’ and ‘UNCERTAIN’. In this study, the ‘UNCERTAIN’ category was assigned to the non-pest action pathway during field testing to enable continuous insect surveying and simplify the autonomous operation of the device. This assignment was an application-specific decision rather than a biological assumption that uncertain species are non-pests. The treatment associated with each classification category can be easily modified within the device control code, allowing users to define different actions for uncertain cases based on the specific application requirements. For example, in pest management applications that require higher confidence, uncertain classifications can be flagged for additional verification, manual review or comparison with user-defined pest databases before determining the final action. This flexible decision framework maintains a three-tier classification structure while allowing for adaptation to different agricultural scenarios.

This AI-driven method enables dynamic prompt adjustment, allowing the classification system to be easily tailored to specific crops, regions or evolving pest management criteria. It provides practical flexibility, allowing users to redefine identification standards as project needs change.

Finally, after the insect has been identified and classified, the main script activates the servo motors to either release or capture/kill the insect. Each observation was recorded in a log and saved to the SD card. The log entry includes the image file, scientific name, common name (if available), first top suggestion scientific name (if available), date and time and action taken by the device, i.e. release or capture. Additionally, it would be feasible to upload logs to be accessed by the user online at regular intervals and delete old observations from the SD card memory. If the primary motivation is to survey or tally insects, the capture function can be disabled so that all insects are released.

### 2.4. Physical design

A Raspberry Pi 4 Model B was picked for its small footprint, which allows for a more compact design of the physical insect catcher. The camera used for insect identification was a Raspberry Pi Camera v1.3. Furthermore, SG90 servo motors were used for the small lightweight design.

The physical device was three-dimensional printed and consisted of a funnel, chamber housing and a killing chamber (figure 2A). The insect enters through the funnel and into the chamber housing, specifically the identification chamber. The camera then views the insect in the chamber, and the iNaturalist identification model and pest dictionary determine if the insect is harmful or not (figure 2B). Harmful insects are moved into the killing chamber, whereas safe insects are released. This is controlled using a servo motor that moves the insect from chamber to chamber (figure 3A).

**Figure 2.**
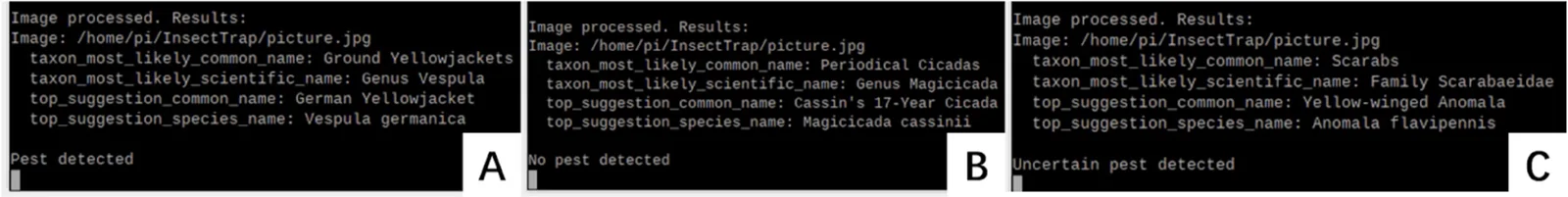
ChatGPT judgement of identified insect type. (A) The identified species ‘Genus *Vespula*’ is judged as a pest. (B) The identified species ‘Periodical Cicadas’ is judged as a non-pest. (C) The identified species ‘Family Scarabaeidae’ is judged as an uncertain pest because it contains pest and non-pest subspecies.

**Figure 3.**
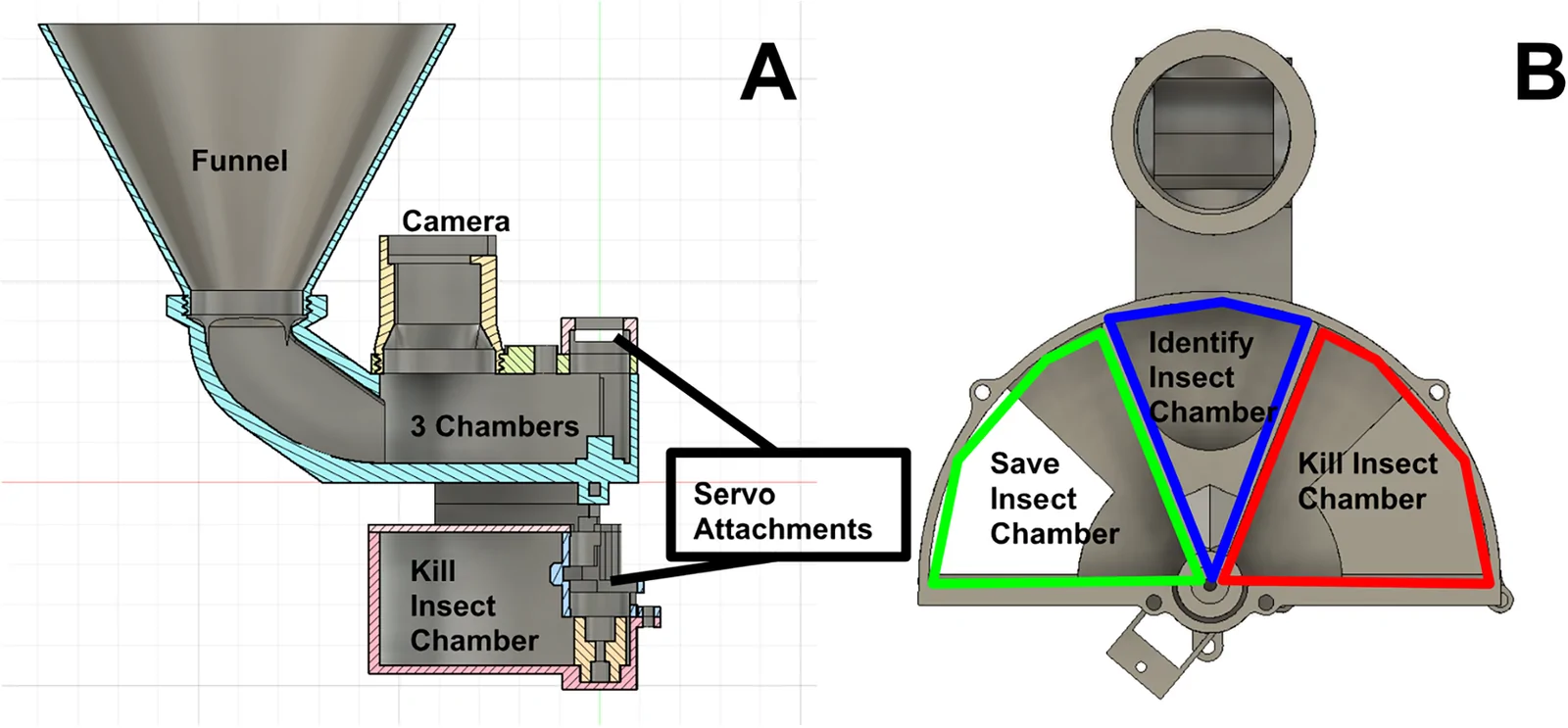
(A) Side view of Insect Catcher. (B) View of the inside compartment. Three chambers are divided by the servo arm. The insect falls into the identify insect chamber via the funnel. The camera then identifies the insect and tells the servo to move it to either the save or kill insect chamber.

Once in the killing chamber, a servo motor begins to crush the insect (figure 4). As of now, hard-shelled insects, such as certain beetles, have the potential to survive. However, a stronger motor would resolve this issue at the expense of compactness.

The funnel was modified to attract insects. Attaching an array of UV lights on the inside will help attract insects; however, current iterations of the design are exploring using pheromone-based methods to attract insects. These pheromones were inserted into the funnel to allow attracting insects day and night, whereas UV light was only effective at night.

## 3. Results

### 3.1. Insect identification and subsequent processes

The program was successful at correctly identifying insects from museum samples that entered the device (figure 5). Most insects were identified at the family and genus levels through the workflow shown in figure 6, which is typically sufficient for managing pests. Several images captured only partial segments of the insect's body, e.g. just the wing or abdomen. This helps mimic the testing with live insects, which will most likely not stay in full frame for the duration they are in the chamber. Fortunately, iNaturalist was still able to correctly identify the genus taxon level (figure 5B,G). Furthermore, three overhead white LED lights were used to illuminate the chamber, which minimized shadows by providing light from multiple directions. This is necessary to allow a clear image to be passed to iNaturalist.

**Figure 4.**
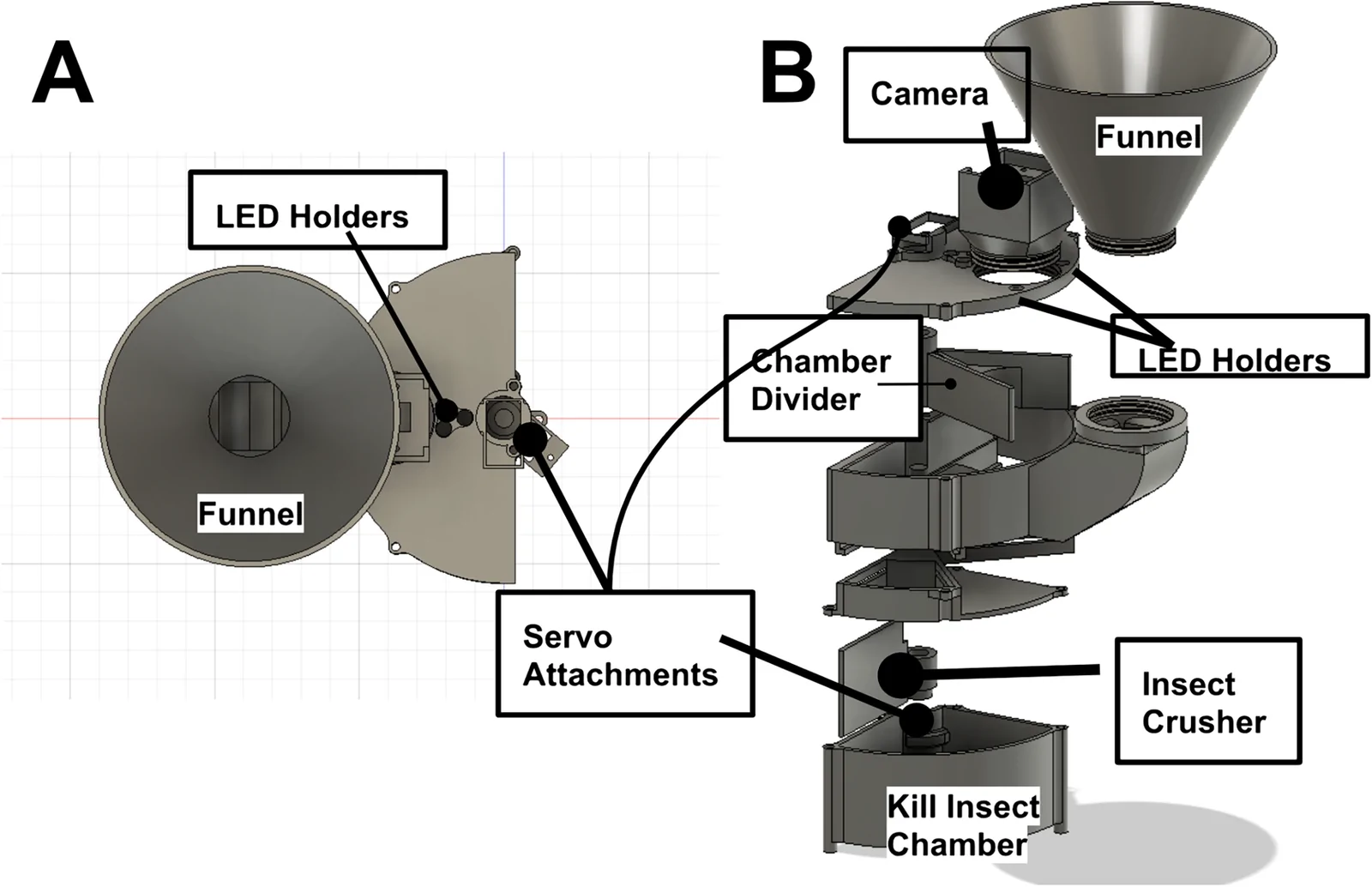
(A) Top-down view of Insect Catcher. (B) Expanded view of Insect Catcher. All components are shown and labelled.

**Figure 5.**
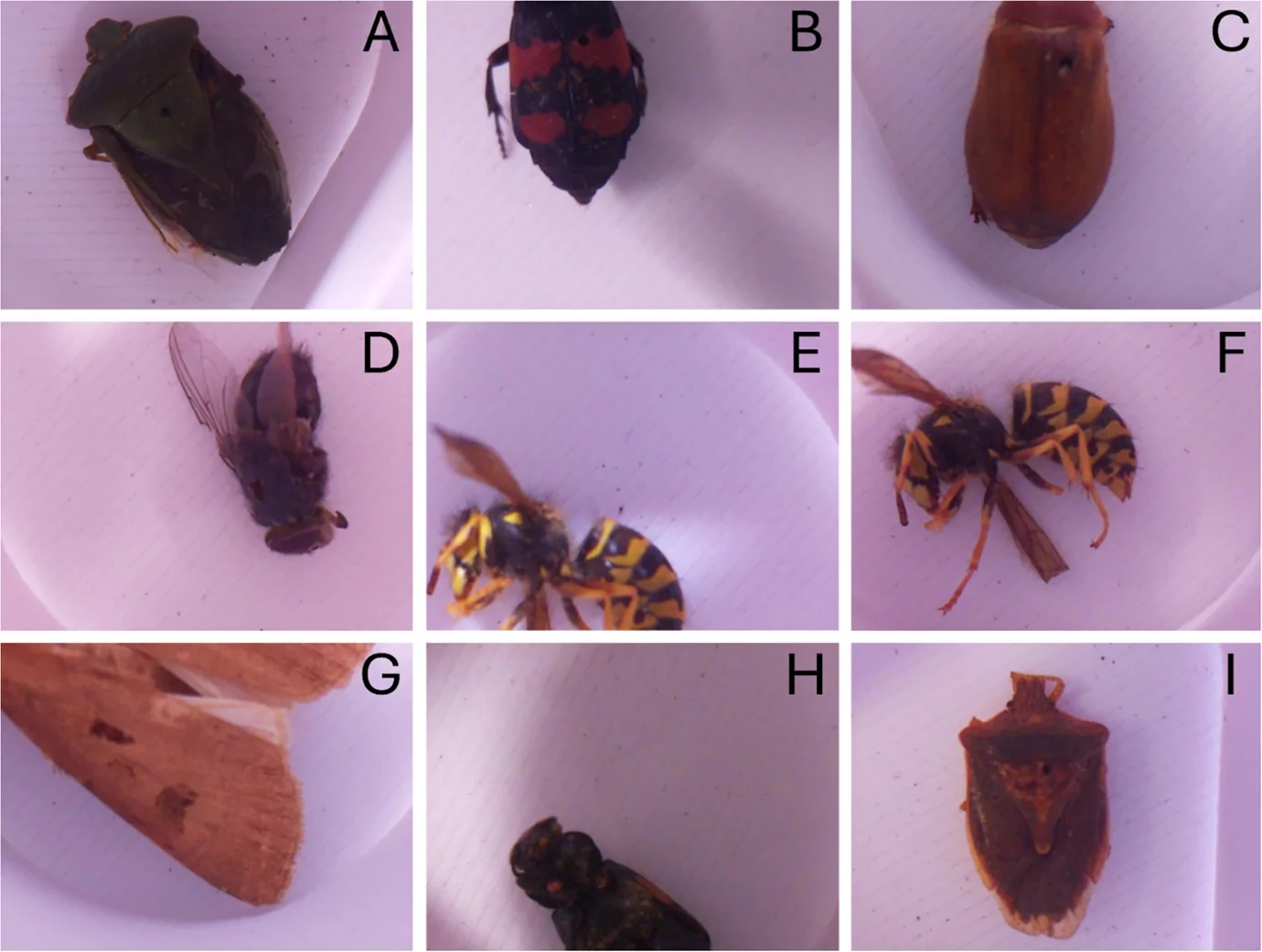
Identification results of images by iNaturalist. All images, except one, were classified correctly at the family taxon level or lower. (A) ‘Family Pentatomidae’. (B) ‘Genus *Nicrophorus*’. (C) ‘Family Scarabaeidae’. (D) ‘Genus *Calliphora*’. (E) ‘Subfamily Vespinae’. (F) ‘Genus *Dolichovespula*’. Note, with a better image compared with (E), a lower-level taxon identification was given. (G) ‘Genus *Agrotis*’. (H) ‘Not Found’. Note, this insect is the same as in (B); however, it is on its back, likely an image with few training examples. (I) ‘Genus *Euschistus*’.

**Figure 6.**
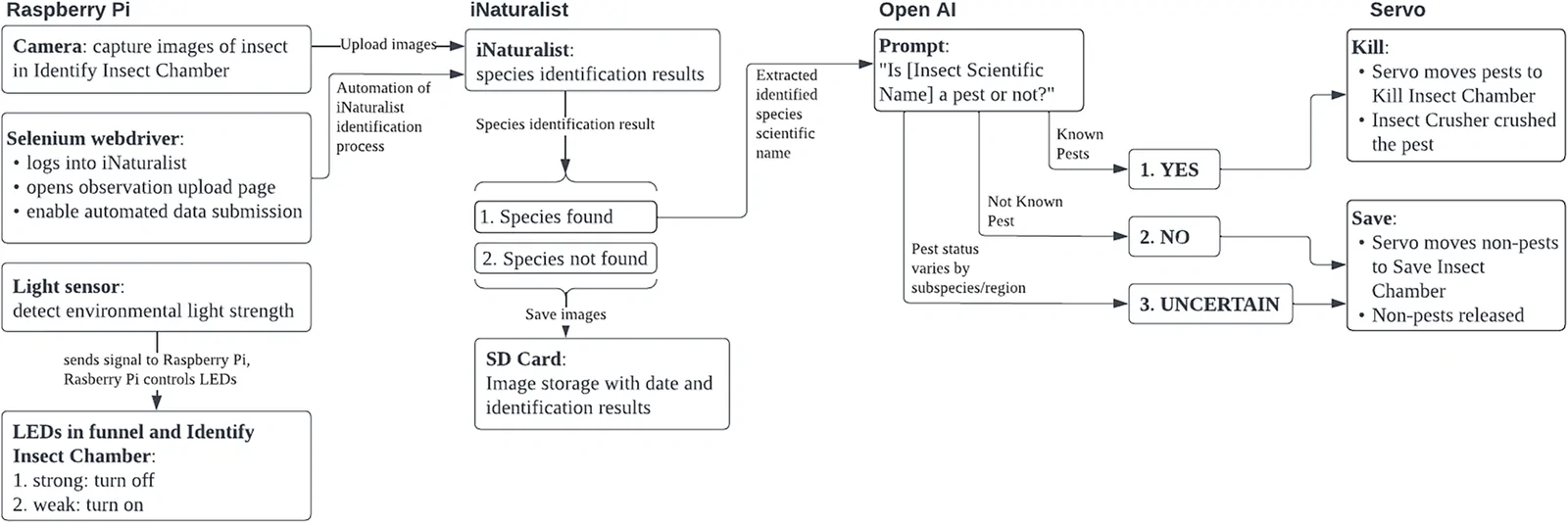
Hardware and software workflow for the pest identification and classification process.

Some improvements are needed to optimize the program. Accessing iNaturalist takes several seconds and requires a steady internet connection during this process. The iNaturalist output is either the identification of the insect or ‘Not Found’. The ‘Not Found’ output results in the release of the insect. When an insect is identified as a pest, it enters the kill chamber. The kill chamber uses a SG90 micro servo for the crushing mechanism (figure 7); therefore, some insects with hard shells (i.e. beetles) may survive the crushing process. Insects that have been tested and successfully crushed include stink bugs, wasps and house flies. Once an insect was deemed a pest, the servo quickly pushed it into the killing chamber. The servo moves quickly enough to ideally throw the insect into the killing chamber without it escaping or clinging onto the walls.

**Figure 7.**
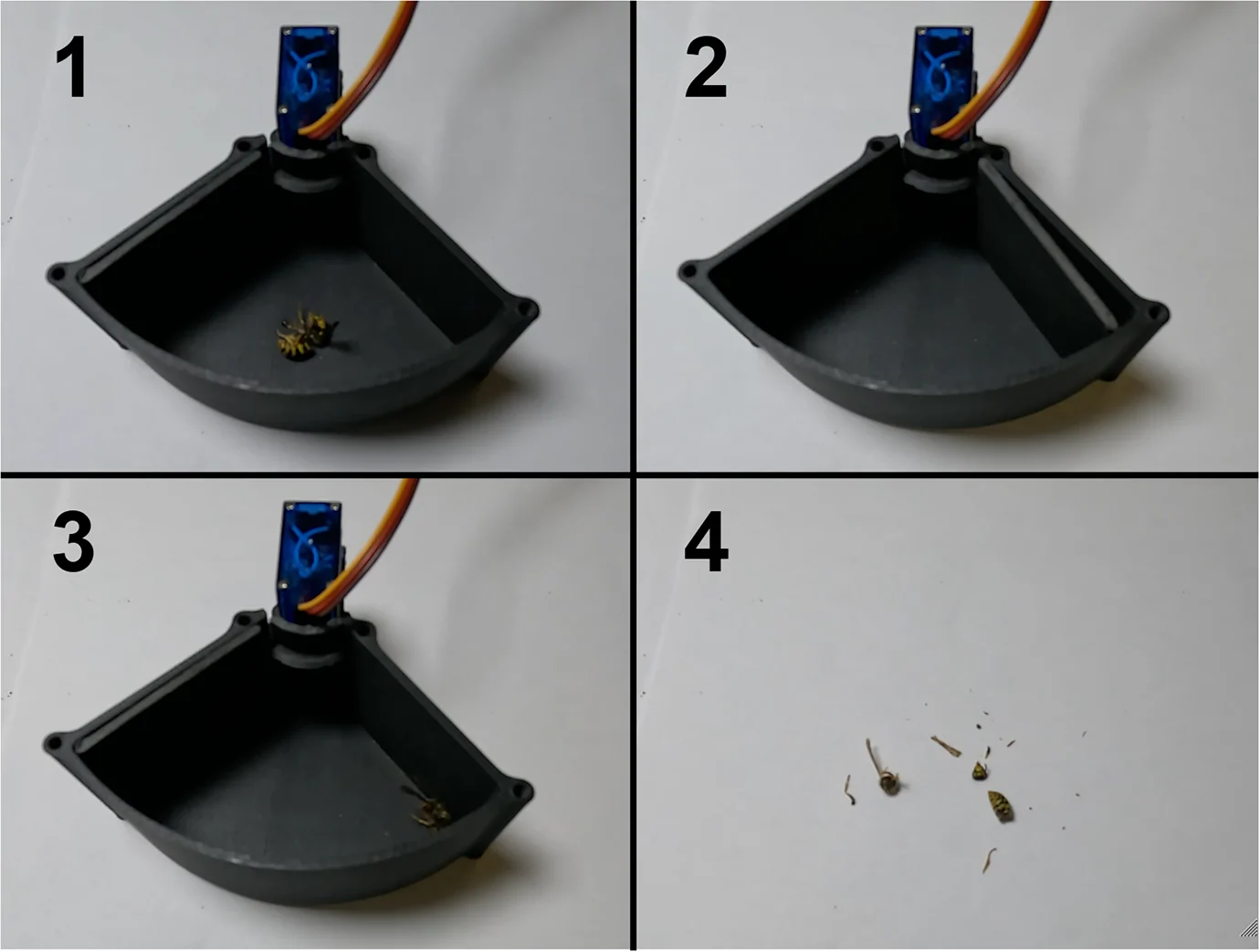
Sequence of a wasp getting crushed in the kill chamber.

**Figure 8.**
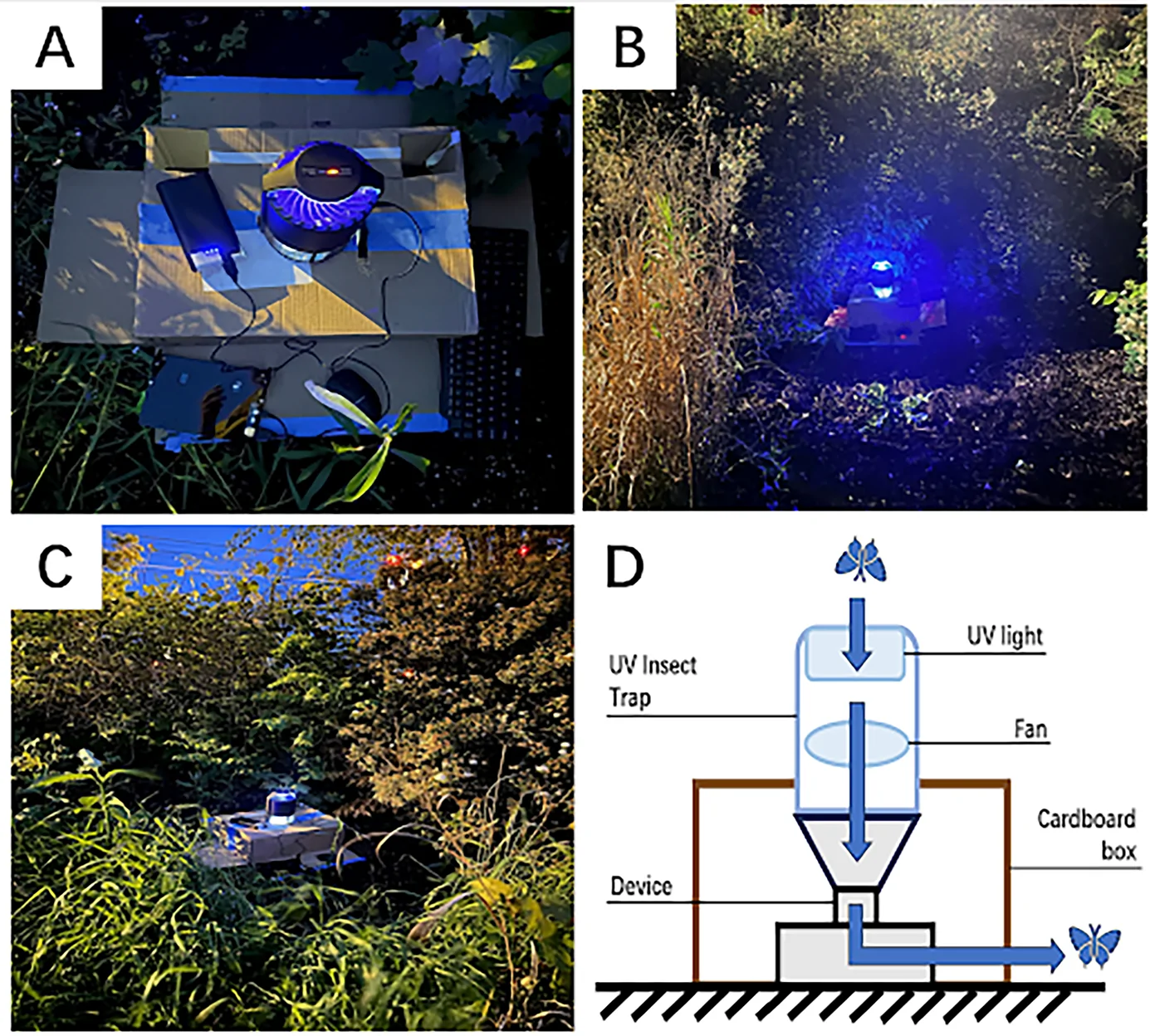
Field trial set-up—UV insect trap mounted on a cardboard box, beneath which is the device. (A) Onsite set-up—top view. (B) Onsite set-up—side view. (C) Onsite set-up—distant view. (D) Schematic diagram.

### 3.2. Field testing

A field test was conducted in July 2025 over 4 days and 2 nights at latitude 42.435°N, longitude 76.495°W. The field test used a charged battery with no solar panel; however, it did show that the physical design was fit for its purpose, being able to last under outdoor conditions for long periods of time. The addition of a solar panel could allow the device and program to run for significantly longer without intervention.

To increase the efficiency of attracting and capturing insects, a commercially available UV insect trap with an inside fan was used, as shown in figure 8. The UV insect trap's collection chamber was opened and the trap was mounted onto a cardboard box, while the device's funnel equipped with LEDs was positioned beneath. This set-up allowed insects to get attracted by the light to be sucked in by the fan, drawn through the funnel and subsequently directed into the insect identification chamber.

Based on the images saved on the Raspberry Pi, images that were blurry or only showed partial insect bodies lacking sufficient diagnostic features for species identification were first filtered out. The remaining images were organized by the corresponding species’ scientific names and categories into three groups: correctly identified, misidentified and insect present, but classified as ‘Not Found’ by the system. For each identified species, the number of images in each category is presented in table 4 in the supplementary material and the corresponding percentages relative to the total number of images for each species are shown in table 5 in the supplementary material.

The identification results suggest a correlation between identification accuracy and the insect average size. For each insect, the percentages of correct identification, misidentification and not-found cases were calculated by dividing the number of images in each category by the total number of images. As multiple species may share a similar average size, a single average size can correspond to several percentage values per category. To visualize this, the mean of these percentage values was used as the representative point for each size, forming the basis for connecting the data points across different sizes. The mean per size data points were used to draw dashed lines, whereas all points were used to compute the linear regression lines.

Based on the linear regression lines, as the average size of the insect samples increased, the percentage of correct identification tended to rise, while the percentage of not-found decreased. The percentage of misidentification oscillates randomly, which may be attributed to certain species being more easily confused with visually similar insects and the specific characteristics shown in the image being taken.

The insect images were categorized into four categories based on the identification result and actual result, and the total number of each category was counted. In total, 719 images were evaluated to assess the accuracy of the insect identification system of the device. The images were classified into four categories:

— True positive when (i) image was identified as correct species (170 images) and (ii) no insect was present or insufficient characteristics, identified as ‘Not Found’ (460 images),
— False positive when the image has no insect present or insufficient characteristics, identified as ‘Found’ and certain species (8 images),
— True negative when image was identified as wrong insect species (14 images), and
— False negative when the image has insects present, identified as ‘Not Found’ and no species (67 images).

Combined, true positives for correct identification and correctly not-found cases accounted for 87.6% of the total, indicating relatively high identification accuracy under the current system, as shown in figure 9.

**Figure 9.**
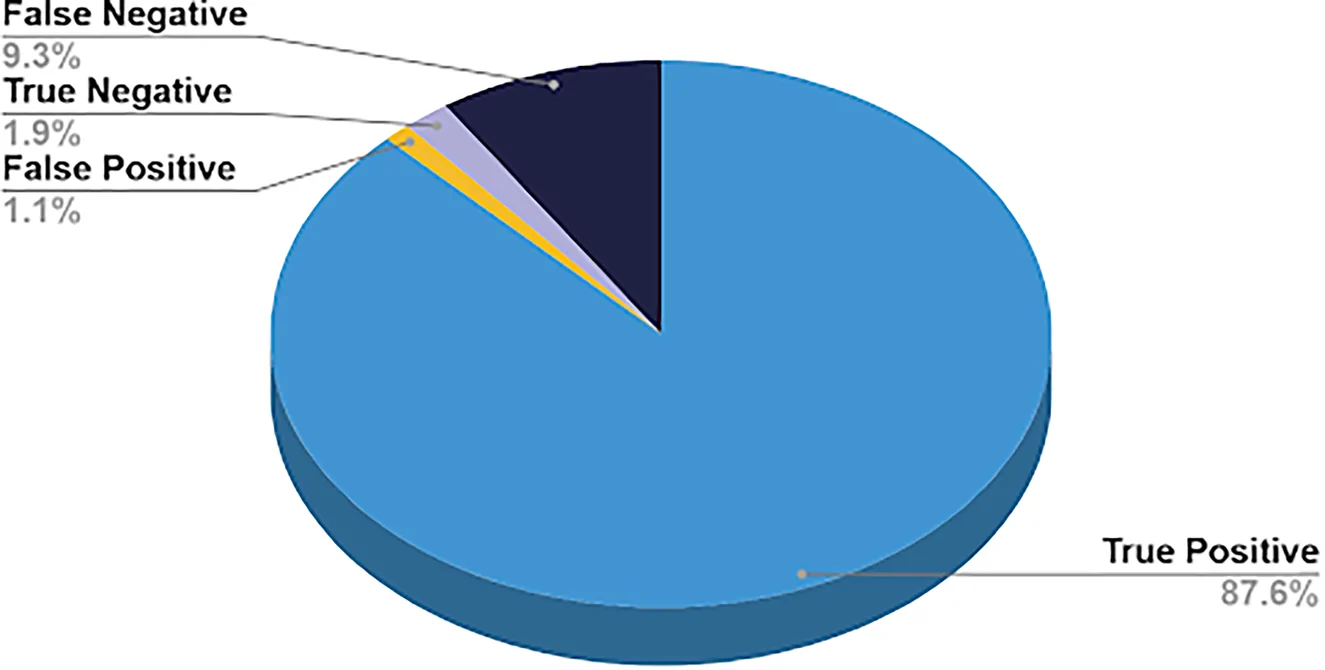
Proportion of the four identification result categories: True positive. False positive. True negative. False negative.

To quantitatively evaluate the performance of our insect identification system, we computed standard classification metrics derived from the confusion matrix, including accuracy, precision, recall and F1‑score. These metrics are essential for assessing the system's performance beyond visual inspection of the prediction results:

$$\begin{array}{rcl} accuracy\; & = & \;\;\frac{TP+TN}{TP+TN+FP+FN}=\;\;\frac{170+460+14}{719}=\;0.896,\\ precision\; & = & \;\frac{TP}{TP+FP}\;=\;\;\frac{170+460}{170+460+8}=\;0.987,\\ recall\; & = & \;\frac{TP}{TP+FN}=\frac{170+460}{170+460+67}\;=\;0.904\\ \text{and}\;\;F1-score\; & = & \;2\times \;\frac{precision\times recall}{precision+recall}=\;2\times \;\frac{0.987\times 0.904}{0.987+0.904}=\;0.944. \end{array}$$

Based on 719 images processed by the device, our system achieved an overall accuracy of approximately 89.6%, indicating that the majority of predictions were correct. The precision was approximately 98.7%, demonstrating that the system made a few false-positive identifications. The recall was approximately 90.4%, showing that most true pest instances were correctly identified. The F1‑score was approximately 94.4%, reflecting a balanced performance between precision and recall.

To calculate the confidence interval (CI) for the performance metrics of our insect identification system, we begin by calculating the standard error (SE) for each metric. We computed for a 95% confidence level:

$$\begin{array}{rcl} SE & = & \sqrt{\frac{p\left(1-p\right)}{n}},\\ CI & = & \;p\pm Z\times SE,\\ Z & \approx & 1.96\;for\;95\%\;confidence,\\ for\;accuracy,\;SE_{accuracy} & = & \;\sqrt{\frac{0.896\left(1-0.896\right)}{719}}\;=\;0.011,\\ \;CI_{accuracy} & = & \;0.896\pm 1.96\times 0.011\;=\;\left[0.874,0.918\right],\\ for\;precision,\;SE_{precision} & = & \;\sqrt{\frac{0.987\left(1-0.987\right)}{719}}\;=\;0.0028,\\ CI_{precision} & = & \;0.987\pm 1.96\times 0.0042\;=\;\left[0.979,0.995\right],\\ for\;recall,\;SE_{recall} & = & \sqrt{\frac{0.904\left(1-0.904\right)}{719}}\;=\;0.0112,\\ CI_{recall} & = & \;0.904\pm 1.96\times 0.0112\;=\;\left[0.882,0.926\right]\\ and\;for\;F1-score,\;SE_{F1} & = & \sqrt{\frac{0.944\left(1-0.944\right)}{719}}\;=\;0.0086,\\ CI_{F1}\; & = & \;0.944\pm 1.96\times 0.0086\;=\;\left[0.927,0.961\right]. \end{array}$$

The accuracy of the model, with 95% confidence, is between 87.4 and 91.8%, confirming its high reliability, with the observed 89.6% accuracy well within a strong range for real world applications. The 95% CI for precision indicates that the model is likely to maintain high precision between 97.9 and 99.5% in future applications, reflecting its effectiveness in minimizing false positives. The recall CI of 88.2 to 92.6% demonstrates the model's strong ability to detect pests, although there remains a slight chance of missing a small percentage of true positives (false negatives). The F1-score's CI, between 93.5 and 95.3%, highlights the model's balanced performance, combining high precision and recall. Overall, these CIs confirm that the model is performing reliably with high precision and good recall, ensuring that it is robust and well suited for practical, real world pest identification applications, with minimal risk of misclassification.

The insect images with correct identification results (true positive) typically display the insect's full body from a clear angle with distinct morphological features, as shown in figure 10. In cases where only partial features are visible, correct identification is still often achieved if the most characteristic traits of the species are captured, such as a uniquely patterned wing or antenna structures.

**Figure 10.**
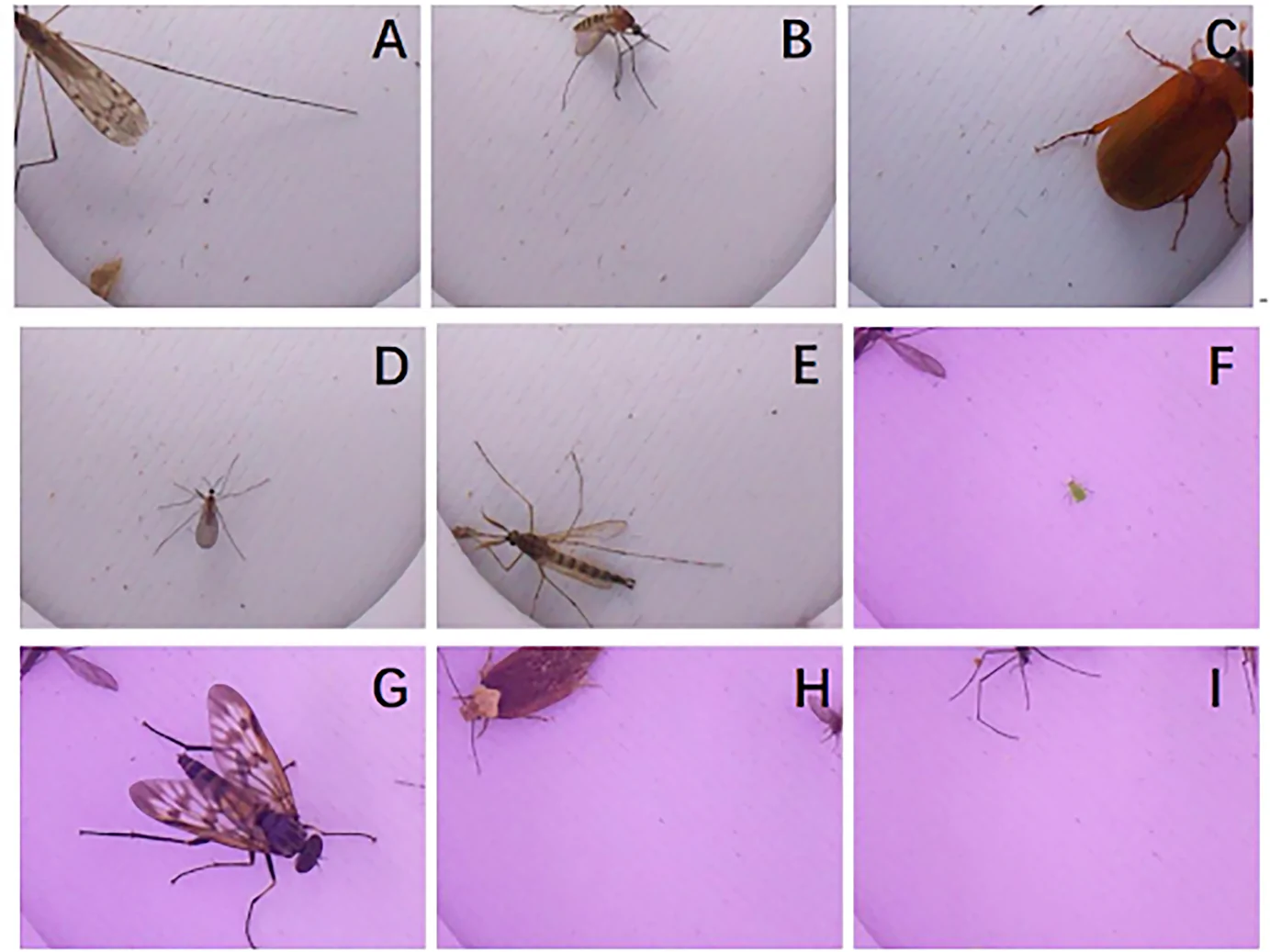
True positive: insect images with correct identification results. (A) ‘Family Limoniidae’. (B) ‘Genus *Culex*’. (C) ‘Tribe Sericini’. (D) ‘Genus *Monarthropalpus*’. (E) ‘Genus *Aedes*’. (F) ‘Subfamily Aphidinae’. (G) ‘Genus *Rhagio*’. (H) ‘Genus *Depressaria*’. (I) ‘Subfamily Culicine’.

Misidentification (true negative) and not-found cases (false negative) commonly occur when the insect is too small in the image, the viewing angle obscures key features, only a portion of the insect is visible or multiple insects are present, which could confuse the models, as shown in figures 11 and 12. The percentage of misidentification may be particularly high for certain species that are difficult to distinguish visually. For example, those that closely resemble other species or require internal morphological features for accurate identification.

**Figure 11.**
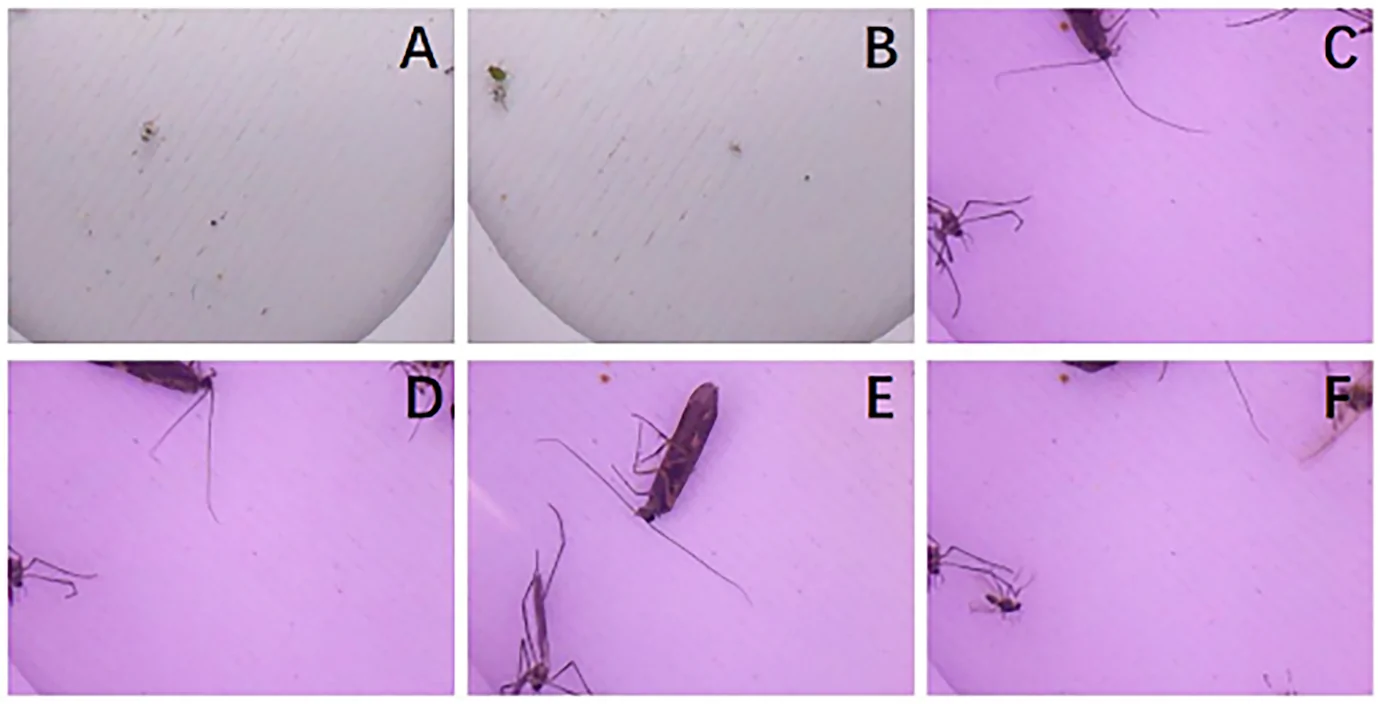
True negative: insect images with misidentification results. (A) ‘Genus *Stephanitis*’ identified as ‘Genus *Anthrenus*’. (B) ‘Tribe Aphidini’ identified as ‘Family Formicidae’. (C) ‘Genus *Mystacides*’ identified as ‘Superfamily Blaberoidea’. (D) ‘Genus *Mystacides*’ identified as ‘Superfamily Gelechioidea’. (E) ‘Genus *Mystacides*’ identified as ‘Genus *Psilochorema*’. (F) ‘Subgenus *Cricotopus*’ identified as ‘Family Veliidae’.

**Figure 12.**
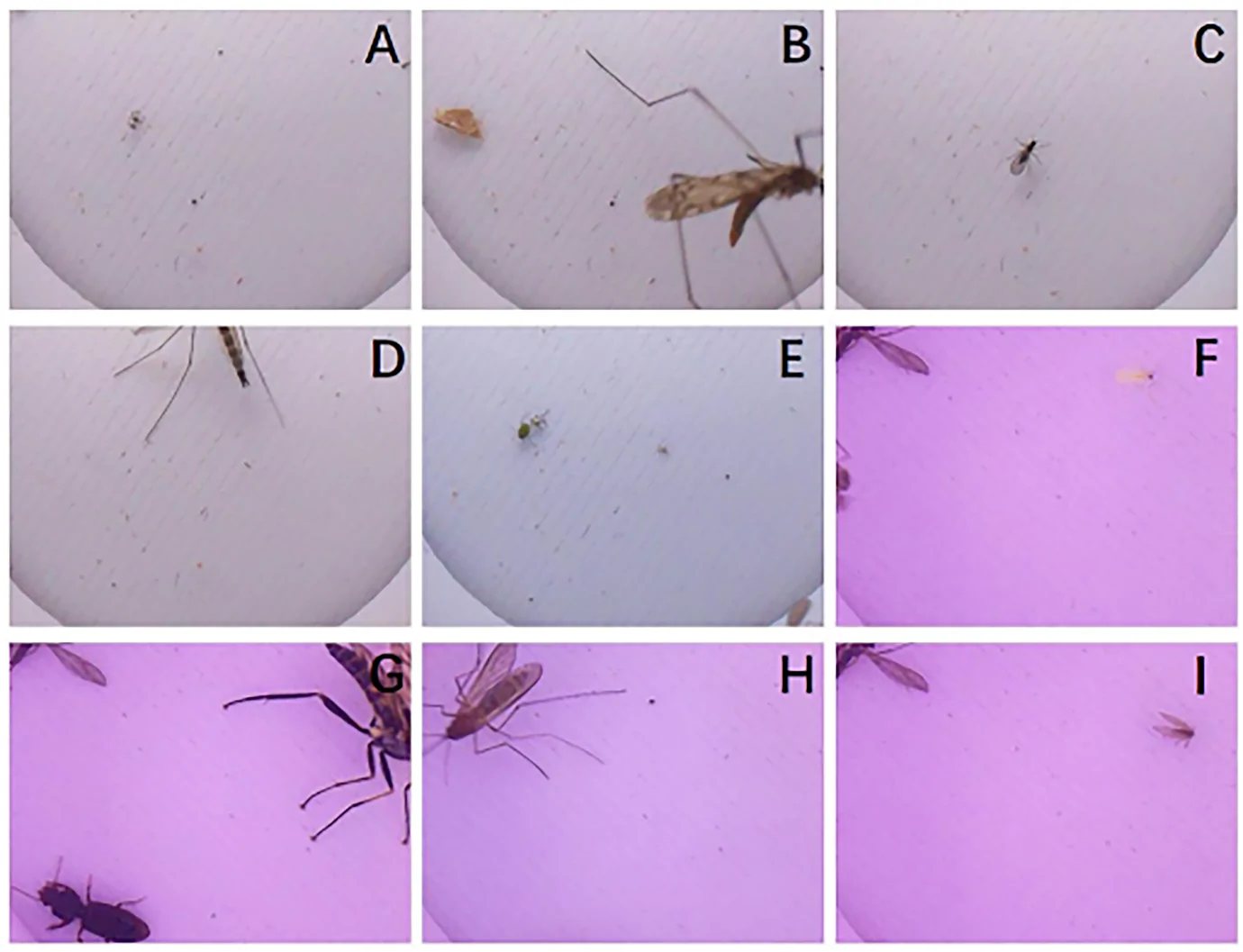
False negative: insect images with relatively sufficient insect characteristics that are identified as (A)–(I) ‘Not Found’.

A relatively smaller portion of images was classified as ‘Found’ with a specific species identified despite lacking sufficient visible insect characteristics (false positive), such as a blank image identified with a present species, as shown in figure 13. This behaviour is expected and may be due to the iNaturalist model being trained on user-submitted observations [21]. Such training data may contain mislabelled or noisy images, leading the model to learn spurious correlations that occasionally result in confident but incorrect identification.

**Figure 13.**
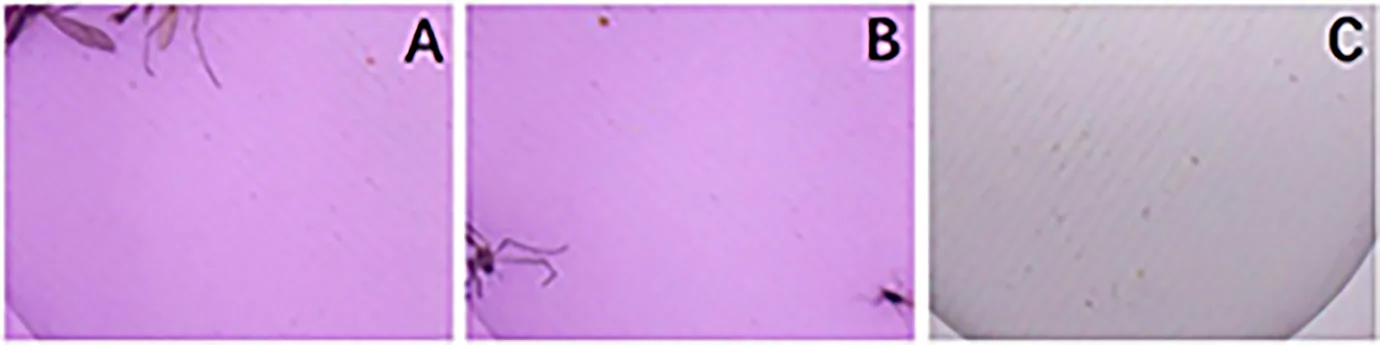
False positive: insect images with insufficient insect characteristics for identification that are categorized as ‘Found’ and have insect species identified. (A) ‘Subfamily culicinae’. (B) ‘Superfamily araneoidea’. (C) ‘Family Formicidae’.

We conducted a species-specific analysis, including accuracy, precision, recall and F1 score, with results shown in table 6 in the supplementary material. The analysis enabled evaluation of species-dependent factors, including body size and morphological distinctiveness, which influence identification performance.

Based on the size-dependent trend observed in figure 14, insect species with average body sizes below approximately 4 mm were further examined to better understand factors contributing to reduced identification performance. Consistent with the overall trend, many small insect species exhibited reduced correct identification rates compared with larger insect species. For example, *Mymaridae* (0.65 mm), *Lachesilla* (1.75 mm), *Thaumatomyia notata* (3 mm) and *Stephanitis* (3.5 mm) achieved correct identification rates ranging from 0 to 6.2%, whereas *Aphidini* (2 mm) achieved a moderate correct identification rate of 40.9%. By contrast, *Monarthropalpus* (2.5 mm) reached a correct identification rate of 88.2% and *Macrosiphini* (3.5 mm) achieved a correct identification rate of 100%, indicating that identification performance is also influenced by factors beyond body size. Species with distinctive morphological characteristics remained identifiable despite their small size. Therefore, the reduced performance observed for some small insects is probably caused by a combination of the small body size and reduced visibility of fine diagnostic features in the captured images under the current imaging conditions. Increasing the camera resolution and improving the chamber illumination through higher-power or additional LED lighting are expected to enhance the visibility of these features and further improve the identification performance, particularly for smaller insect species.

**Figure 14.**
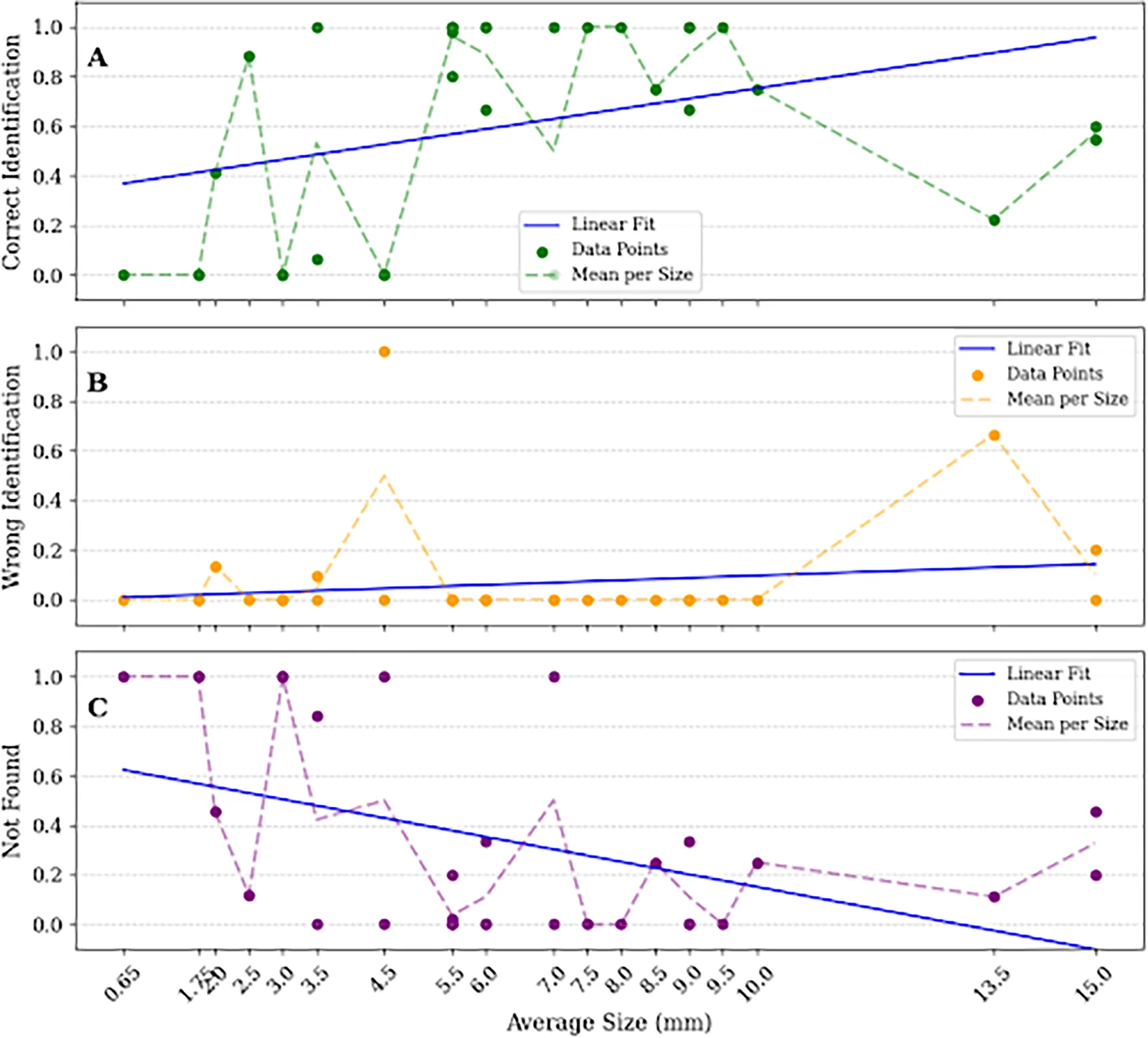
(A) Percentage of correct identification with respect to the average size of each insect. (B) Percentage of wrong identification with respect to the average size for each insect. (C) Percentage of ‘Not Found’ with respect to the average size for each insect.

These results indicate that body size alone cannot fully explain identification performance. Morphological distinctiveness also strongly influenced identification performance across different insect sizes. For example, the *Aedes* genus (5.5 mm), with its distinct wing patterns and body shape, achieved a correct identification rate of 97.7% with nearly perfect precision and recall. Similarly, *Monarthropalpus* (2.5 mm) and *Trentepohlia* (15 mm), which exhibit clear morphological characteristics, achieved correct identification rates above 80%, demonstrating the model's ability to recognize species with distinctive visual features. By contrast, species with visually similar appearances or limited diagnostic features, such as *Stephanitis* (3.5 mm), showed reduced identification performance. Taxa such as *Formicidae* (ants) and *Araneoidea* (spiders), which were occasionally predicted as alternative categories, further highlight the remaining challenges in identifying visually similar taxa.

### 3.3. General AI pest identification

A random sample of 1000 insect species was selected from the 6019 species listed in the most recent U.S. Regulated Plant Pest Table released by the U.S. Department of Agriculture (USDA) [22]. This sample size ensured a 95% confidence level with a ±3% margin of error, providing a statistically reliable and robust assessment of the tool's accuracy, suitable for both external reporting and academic review. Since the USDA list of insect pests does not include pests that are present and may be widely distributed in the USA, we also gathered 262 US-regulated pest species names from other authoritative sources, including the APHIS Pest and Disease Lists and the Pest Tracker (National Agriculture Pest Information System) [23]. Additionally, we retrieved 20 common beneficial insect species from the USDA National Resource Conservation Service database [24].

For both pest species and beneficial insects, we used the following prompt for classification: ‘Is [Insect Scientific Name] a pest or not in the USA? Answer me only with YES, NO (NO if the species is both a pest and a beneficial insect).’ Note that the response ‘UNCERTAIN’ was excluded from the analysis, as the list includes specific insect species names rather than broader categories (which may sometimes result from species identification platforms like iNaturalist). The responses from ChatGPT were then collected and analysed to evaluate the model's accuracy in identifying regulated pests and distinguishing them from beneficial species. The results of this evaluation are shown in table 1 and detailed information is provided in the supplementary material.

The analysis of pest and non-pest classification by ChatGPT reveals its overall effectiveness and accuracy. Based on the data provided, a confusion matrix was constructed to evaluate the model's performance in identifying pests and non-pests. The model correctly identified 1087 pests (true positives, TP) and misclassified 175 pests as non-pests (false negatives, FN). It also accurately classified 19 non-pests (true negatives, TN) and mistakenly identified 1 non-pest as a pest (false positive, FP).

The overall accuracy of the model is 86.3%, indicating that it correctly classified pests and non-pests 86.3% of the time. Precision, which measures the accuracy of pest predictions, is exceptionally high at 99.9%, meaning that nearly all species predicted as pests are indeed pests. However, recall for pests was slightly lower at 86.1%, indicating that the model missed approximately 13.9% of actual pests. The F1-score, which balances precision and recall, is 92.3%, demonstrating a strong overall performance in pest identification. On the non-pest side, the model performed well in terms of specificity, correctly identifying 95% of non-pests, but the false-positive rate was 5%, meaning that 5% of non-pests were incorrectly classified as pests. This suggests that the model is slightly more prone to identifying non-pests as pests but still does a good job at distinguishing between the two categories.

In summary, the ChatGPT pest identification model demonstrates high precision and specificity, making it a reliable tool for pest identification. However, there is room for improvement in its recall, particularly in reducing false negatives where certain pests may be overlooked. Given the inherent limitations and potential biases of ChatGPT, this model could be enhanced by integrating it with authoritative pest databases, such as those maintained by the USDA, Food and Agriculture Organization (FAO) or local pest lists. By combining these resources, the accuracy of the model can be significantly improved for specific use cases. Additionally, the model's prompt can be tailored to address particular pest categories or regions, further optimizing its performance. Overall, while the model is a strong starting point, further refinement and integration with external pest data would increase its effectiveness and suitability for pest management and classification tasks.

## 4. Discussion

Compared with traditional methods of insect identification, our method of leveraging existing cloud platforms with built-in models streamlines the process, making it more accessible and offering a more efficient and accurate alternative. This approach directly benefits from the extensive iNaturalist research-grade observations used to train the CV model through frequent update cycles. Previous approaches to developing this insect catcher focused on training a small CV model that would perform inference locally on the Raspberry Pi; however, this approach was difficult to scale and could not match the accuracy or species scope of the iNaturalist identification and ChatGPT classification systems. There is also a similar computational load between using a web driver and running a small local model for inference. The overall workflow can also be viewed as an agentic-AI-like approach in which multiple AI-enabled tools are coordinated to complete different stages of the task. Rather than relying on a single model, the system uses browser-based automation to obtain visual identification from iNaturalist and then uses ChatGPT for pest classification. This modular design allows individual components to be modified or replaced as improved AI tools become available, providing flexibility for future system development. In this study, improvements can be made to the iNaturalist and ChatGPT query approaches to generate the identification and classification more quickly.

### 4.1. YOLO-based comparative evaluation

To provide empirical grounding for comparison with a conventional You Only Look Once (YOLO)-based approach, we additionally evaluated the same 512 field-test images using an existing YOLO model trained on 15 predefined insect categories. The YOLO model is a CNN-based object detection model that accepts 416 × 416 × 3 RGB images as input. Its architecture consists of nine convolutional layers, six max-pooling layers and eight Leaky Rectified Linear Unit (ReLU) activation layers, with approximately 11.063 million trainable parameters. The convolutional layers progressively increased the number of feature channels before producing a final 13 × 13 × 100 raw detection tensor. The model was deployed in TensorFlow Lite (TFLite) format with a model size of 21.11 MB. On the test computer, the model achieved an average inference time of 52.26 ms per image, corresponding to approximately 19.13 frames per second. Additional model characteristics are summarized in the supplementary material.

Because many of the field-observed taxa were not included in the YOLO model's training classes, this experiment was designed as a limited comparison of model behaviour on a common set of field images rather than as a direct closed-set accuracy benchmark. A confidence threshold of 0.75 was first used to distinguish between high- and low-confidence predictions. The results are summarized in table 8 in the supplementary material. Overall, 477 of the 512 images (93.16%) exhibited the predefined expected behaviour, whereas 35 images (6.84%) exhibited unexpected behaviour according to the confidence and taxonomic criteria defined in table 2.

**Table 1.** ChatGPT identification of insect pest and beneficial species in the USA.

insect species list	number of species	number of identified pests	number of identified non-pests
USDA insect pest list	1000	831	169
pest tracker list	262	256	6
common beneficial insect list	20	1	19

**Table 2.** Empirical comparison of YOLO-based classification behaviour on field-test images.

outcome	definition	confidence threshold 0.5	confidence threshold 0.75
		number of images/percentage	number of images/percentage
**expected behaviour**	1. high-confidence correct prediction for included taxon 2. low-confidence prediction for unseen/unincluded taxon	406/79.30%	477/93.16%
**unexpected behaviour**	1. low-confidence prediction for included taxon 2. high-confidence prediction for unseen/unincluded taxon	106/20.70%	35/6.84%
**total**		**512/100%**	**512/100%**

When the confidence threshold was lowered from 0.75 to 0.50, the proportion of images exhibiting expected behaviour decreased from 93.16 to 79.30%, whereas the proportion exhibiting unexpected behaviour increased from 6.84 to 20.70%. This decrease indicates that the observed behaviour of the fixed-category YOLO model was sensitive to the selected confidence threshold. Although the 0.75 threshold produced a slightly higher expected-behaviour rate than the 87.6% true-positive percentage observed for the proposed iNaturalist-based system, this apparent advantage was not maintained at the lower 0.50 threshold. These results further demonstrate why the two systems should not be compared solely on the basis of a single confidence threshold or a single performance metric.

These results illustrate a limitation of fixed-category YOLO models when applied to field images containing previously unseen taxa. By contrast, the proposed iNaturalist-based approach is not restricted to a fixed set of locally trained species categories and therefore provides greater flexibility for identifying diverse and previously unmodelled insect taxa. However, because the YOLO model was not trained or evaluated under the same closed-set conditions as the proposed system, and because the two approaches address somewhat different identification settings, the expected-behaviour rates of 93.16 and 79.30% should not be interpreted as direct accuracy comparisons with the 89.6% identification accuracy of the proposed system. Rather, this experiment provides empirical evidence of the behaviour and threshold sensitivity of a conventional fixed-category YOLO model when exposed to the same field-test images used to evaluate the proposed system.

### 4.2. Device and system limitations

One limitation that needs to be addressed is the possibility of more than one insect entering the chamber at once. Currently, the iNaturalist program will either be unsure of the insect (i.e. return ‘Not Found’) or will identify only one of the insects. This is not ideal, as one of these insects could be a pest while the other is not. Another limitation is the simplicity of the entrance funnel. Currently, there is nothing stopping the insect from exiting out of the entrance. Another factor that could increase the chance of the insect exiting is the several seconds of processing time of iNaturalist to identify the insect. To resolve these limitations, a more automated entrance that can close and reopen when an insect enters can be implemented in the future. This will not only close the entrance once an insect enters, but also allow the insect to move along one direction only.

As for iNaturalist, the faster the program is at identifying the insect, the less likely it is to escape from the entrance; therefore, speeding up the identification process is necessary. The reliance on a steady internet connection limits the location and conditions this device can be used. Under these conditions, the internet connection and speed could hinder this device's performance. Also, some locations where this device could be employed may not have a steady internet connection as well. Unfortunately, this method is still preferred until an on-device insect identification method with performance levels comparable to iNaturalist is developed.

### 4.3. Image quality and identification performance

Several of the identified insects in the results were only partially visible in the images; however, iNaturalist would be able to make better predictions with more of the insect visible. One option is to extend the camera further away from the identification chamber. A simpler option would be to switch the current camera to a wide-angle lens to capture more of the chamber and, thus, more of the insect. Another improvement in classification would be to encode the geolocation of where the image was captured into the image. Currently, the Raspberry Pi camera does not include this information, but it would help in identification, as the iNaturalist CV model also utilizes geographical features in its model.

Field test results indicate that identification accuracy follows a clear trend relative to the average body size of insect species. As the average size increases, the percentage of correct identification rises, while the percentage of not-found cases decreases. Since misidentified images account for only approximately 1.9% of the total dataset, a slight increase in the misidentification percentage among larger insects could be attributed to variations in the sample distribution. Analysis of images further confirmed that the accuracy of insect identification is strongly influenced by image quality, especially the visibility of key morphological features. Full-body images with clear and distinguishing traits significantly increase the likelihood of correct identification. However, poor angles, partial visibility or multiple insects can lead to misclassification or not-found outcomes. Furthermore, 1.1% of the images classified as false positives were confidently identified at the species level, despite lacking sufficient visual information. This highlights a limitation in the model's training data, which relies heavily on user-submitted observations that may contain mislabelled or noisy samples.

Despite these limitations, the device demonstrated strong practical reliability, with 87.6% of cases either correctly identified insect species or appropriately rejected as ‘Not Found’. Notably, the device was able to provide identification results in several cases where multiple individuals appeared in the frame, suggesting potential robustness under more complex visual conditions.

### 4.4. Limitations of iNaturalist and ChatGPT-based pest classification

While iNaturalist provides broad species and geographic coverage, its database may still have limitations, particularly with regard to regionally specific or rare species. Although the platform encompasses a vast array of species across diverse ecosystems, its capacity to capture every local or under-researched pest remains constrained. Nevertheless, iNaturalist remains one of the largest and most widely used citizen science platforms, with over a million users globally, contributing valuable species data [25]. This extensive network allows for continuous updates and the inclusion of a wide variety of species, including those that are specific to local regions. In cases where iNaturalist does not include highly localized species, locally trained deep learning models can be more effective. These models are specifically designed to address environmental conditions, pest behaviours and unique species found within particular regions [26]. Despite the challenges in identifying very local species, our model remains flexible and offers a wide range of coverage, making it suitable for various agricultural contexts.

While ChatGPT shows strong potential for pest identification, it is important to address certain limitations when applying it to pest identification. Training data bias arises from the model's reliance on publicly available English-language sources, which tend to over-represent pest-related information from North America and Europe. This bias may under-represent minor crops, region-specific pests and pests from non-English or developing regions, leading to generalized classifications that may not align with local agricultural practices. Overgeneralization bias occurs when the model simplifies pest identification to a binary decision (e.g. ‘Is [species] a pest in Ithaca, NY?’), overlooking contextual factors such as crop specificity, seasonal effects and pest behaviour. These factors are critical in pest management; however, the binary nature of the prompt could miss the nuances that affect pest classification. Confirmation bias may lead the model to favour plausible conclusions without sufficient data, instead of providing a nuanced assessment. The model may be prone to making absolute decisions based on limited evidence, potentially overlooking the uncertainty. Finally, regional variability is a challenge, as the model struggles to account for location-specific pest behaviours, seasonal changes and updated agricultural policies. Although these biases present challenges, they can be alleviated by refining the ChatGPT prompt to better suit specific use cases by incorporating more detailed descriptions and requirements, such as crop type and updated pest regulation policies. Additionally, combining the ChatGPT pest identification model with official pest lists could enhance accuracy or with local pest lists for improved performance in region-specific applications. Currently, we have not integrated the model with these lists to maintain flexibility, especially since widely distributed pest lists in the USA, such as those from the USDA, focus primarily on invasive species. Future improvements could include combining global and localized data to develop models adapted to specific regions and incorporating local pest lists for more accurate and region-specific applications.

### 4.5. Practical applications and future works

Overall, the physical device integrated with the insect identification/classification program can provide a practical and cheap method for the agricultural industry to remove pests from farms without harming beneficial insect populations. It can also be used as a way to survey insect populations within the farm, which can be useful for population tracking over time. In agriculture, early detection of pests can lead to proactive interventions, potentially mitigating the extensive damage pests often inflict on crops. This not only has economic ramifications but also impacts food security. The integration of our model with a physical design offers a scalable solution for monitoring large agricultural areas, potentially revolutionizing pest control methods. Our team is currently working on improving the insect detection method to enhance the scalability of the device and reduce the reliance on a steady internet connection. Additional field testing is also being conducted at different locations and under varying environmental conditions to further evaluate the robustness and adaptability of the system in diverse agricultural environments.

## Supplementary Material



## Data Availability

The data presented in this study are available at OSF [27]. Supplementary material is available online [28].
