# Supplementary material for: Smart insect trap: insect identification and classification using iNaturalist and ChatGPT
Source: R Soc Open Sci. 2026 Oct 28;13(10):251212. doi: 10.1098/rsos.251212 (PMC13714388; doi:10.1098/rsos.251212)
Supplement: Supplementary file 1 [file rsos.251212.zip › rsos.251212/rsos251212_si_002.pdf]

Appendix A. Initial Setup of Raspberry Pi Environment

Start with a new/fresh Raspberry Pi OS image to the SD card. Our device used Raspberry Pi 4 OS (64-bit) and was loaded onto the SD card with Raspberry Pi Imager, https://www.raspberrypi.com/software/.

Follow instructions to set up Raspberry Pi and connect to the internet. Our device used default initial settings., https://www.raspberrypi.com/documentation/computers/getting-started.html.

Open the terminal / command shell to update packages.

```
sudo apt update
sudo apt upgrade
```

Install git package.

```
sudo apt install git
```

Download InsectTrap code in the directory of your choice, e.g. `/home/pi`.

```
git clone https://github.com/anthonynic28/InsectTrap.git
```

Install Pyenv, https://github.com/pyenv/pyenv, for the Insect Trap virtual environment.

```
curl https://pyenv.run | bash
```

Set up your shell environment for Pyenv.

```
echo 'export PYENV_ROOT="$HOME/.pyenv"' >> ~/.bashrc
echo 'command -v pyenv >/dev/null || export PATH="$PYENV_ROOT/bin:$PATH"' >>
~/.bashrc
echo 'eval "$(pyenv init -)"' >> ~/.bashrc
```

Restart shell.

```
exec $SHELL
```

Install Python dependencies.

```
sudo apt install --yes libssl-dev zlib1g-dev libbz2-dev libreadline-dev
libsqlite3-dev llvm libncurses5-dev libncursesw5-dev xz-utils tk-dev
libgdbm-dev lzma lzma-dev tcl-dev libxml2-dev libxmlsec1-dev libffi-dev
liblzma-dev wget curl make build-essential openssl
```

Update Pyenv package.

```
pyenv update
```

Install python 3.11.2 to Pyenv.

```
pyenv install 3.11.2
```

Create a new virtual environment with Python 3.11.2 within the InsectTrap folder you cloned from git.

```
cd InsectTrap
pyenv virtualenv 3.11.2 insect-venv
```

Activate the new virtual environment.

```
source .insect-venv/bin/activate
```

Update pip package while insect-venv is active.

```
python -m pip install --upgrade pip
```

Install InsectCatcher dependencies, modify path to requirements.txt if necessary.

```
python -m pip install -r /home/pi/InsectTrap/requirements.txt
```

Start pigpio daemon to control servo motors before running Python script.

```
sudo pigpio
```

All necessary packages should be installed and InsectTrap can be initialized with auto mode off. Note, it will be necessary to add your own username and password in the `Get_Classification.py` script to the variables: `username` and `password`.

```
python InsectTrapProject/main.py --autoOFF
```

Appendix B. Prototype Cost

Overall, the device itself cost $24.44 USD with the solar panel and solar battery manager costing $45.99 USD (Table 2). The cost of PLA plastic used for 3D printing was not included in the cost analysis.

Table 2. Cost and quantity of the components for the insect trap. Cost of 3D printed PLA not included.

Item	Quantity	Price/each
Raspberry Pi 4 Model B	1	$35.00
SG90 micro Servo	2	$1.90
White LED	3	$0.06
Adafruit BH1750 Ambient Light Sensor	1	$4.50
Jumper wires	16	$0.06
**Subtotal**		**$41.52**
20W Solar Panel	1	$20
WaveShare Solar Power Manager (B)	1	$25.99
**Full Total**		**$87.51**

Appendix C. Field Test Insect Identification Result Tables

After filtering out images lacking sufficient information for insect identification, the remaining images for each species were categorized into Correct Identification, Misidentification, and Not Found. Table 3 presents, for each species, the scientific name, the number of correctly identified images along with their identified species names, the number of misidentified images with corresponding species names, and the number of Not-Found images. Based on the data in Table 3, the percentage of images in each category relative to the total number of images for each species was calculated and is presented in Table 4. Based on the data in Table 4, the species-specific confusion matrix analysis, including precision, accuracy, recall, and F1 score, is presented in Table 5. "/" indicates that the computation could not be performed due to a zero in the numerator of the confusion matrix calculation.

Table 3. Image Identification Results for each Species of Insect Trapped by the Device

Species Scientific Name	Identified Correct	Identified Wrong	Not Found	No Insect present but Found
Genus Stephanitis	2	2（Genus Anthrenus）<br>1 (Superfamily Miroidea)	27	
Tribe Sericini	1			
Tribe Aphidini	3 (Tribe Aphidini)<br>6 (Subfamily Aphididae)<br>1 (Family Aphididae)	3 (Family Formicidae)	10	
Family Mymaridae			2	
Lachesilla Pedicularia			3	
Genus Nipponoserica	1 (Family Scarabaeidae)<br>1 (Tribe Sericini)<br>1 (Genus Nipponoserica)		1	
Genus Trentepohlia	1 (Genus Trentepohlia)<br>1 (Family limoniidae)<br>1 (Superfamily Tipuloidea)	1 (Genus Antocha)	1	
Superfamily Tipuloide	6		5	
Genus Monarthropalpus	14 (Genus Monarthropalpus)<br>1 (Tribe Eriopterini)		2	
Tribe Lathrobiiini	4		2	
Tribe Macrosiphini	2 (Tribe Macrosiphini)<br>3 (Subfamily Aphidinae)			
Genus lachesilla			1	
Genus Mycomya	1			
Superfamily Culicinae	3			2

Species Scientific Name	Identified Correct	Identified Wrong	Not Found	No Insect present but Found
Genus Aedes	19 (Genus Aedes)<br>3 (Tribe Aedes)<br>21 (Subfamily Culicinae)		1	
Genus Rhyssemus	1			
Genus Staphylinidae	1			
Subfamily Chironominae	3			
Genus Rhagio	39			
Genus Culex	3 (Genus Culex)<br>1 (Subfamily Culicinae)		1	
Superfamily Muscoidea	2			
Genus Depressaria	4			
Superfamily Sciaroidea	1			
Genus Molophilus	2			
Subtribe Chironomus	1			
Family Limoniidae	3		1	
Genus Mystacides	2	1 (Genus Psilochorema)<br>3 (Superfamily Blaberoidea)<br>2 (Superfamily Gelechioidea)	1	
Genus Anopheles	2		1	
Subgenus Cricotopus		1 (Family Veliidae)		
Genus Paraproba			1	
Clivina Fossor			2	
Thaumatomyia notata			2	
Psychoda alternata			1	
Thaumatomyia notata			1	
Family Formicidae				4
Subfamily Araneoidea				2

Table 4. Image Identification Percentages for each Species of Insect Trapped by the Device

Species Scientific Name	Average Size (mm)	Identified Correct	Identified Wrong	Not Found
Family Mymaridae	0.65	0	0	1
Lachesilla Pedicularia	1.75	0	0	1
Genus lachesilla	1.75	0	0	1
Tribe Aphidini	2	0.409	0.136	0.455
Genus Monarthropalpus	2.5	0.882	0	0.118
Thaumatomyia notata	3	0	0	1
Psychoda alternata	3	0	0	1
Thaumatomyia notata	3	0	0	1
Genus Stephanitis	3.5	0.062	0.094	0.844
Tribe Macrosiphini	3.5	1	0	0
Subgenus Cricotopus	4.5	0	1	0
Genus Paraproba	4.5	0	0	1
Genus Culex	5.5	0.800	0	0.200
Superfamily Culicinae	5.5	1	0	0
Genus Aedes	5.5	0.977	0	0.023
Genus Rhyssemus	5.5	1	0	0
Superfamily Sciaroidea	5.5	1	0	0
Genus Molophilus	5.5	1	0	0
Genus Staphylinidae	6	1	0	0
Subfamily Chironominae	6	1	0	0
Genus Anopheles	6	0.666	0	0.333
Clivina Fossor	7	0	0	1
Genus Mycomya	7	1	0	0
Superfamily Muscoidea	7.5	1	0	0
Subtribe Chironomus	8	1	0	0
Genus Nipponoserica	8.5	0.750	0	0.250
Genus Rhagio	9	1	0	0
Tribe Sericini	9	1	0	0
Tribe Lathrobiiini	9	0.667	0	0.333
Genus Depressaria	9.5	1	0	0

Species Scientific Name	Average Size (mm)	Identified Correct	Identified Wrong	Not Found
Family Limoniidae	10	0.750	0	0.250
Genus Mystacides	13.5	0.222	0.667	0.111
Genus Trentepohlia	15	0.600	0.200	0.200
Superfamily Tipuloide	15	0.545	0	0.455

Table 5. Species-Specific Confusion Matrix Analysis for Insect Species Identification

Species Scientific Name	Accuracy	Precision	Recall	F1-Score
Genus Stephanitis	0.15625	1	0.068966	0.129032
Tribe Sericini	1	1	1	1
Tribe Aphidini	0.565217	1	0.5	0.666667
Family Mymaridae	0	/	0	/
Lachesilla Pedicularia	0	/	0	/
Genus Nipponoserica	0.75	1	0.75	0.857143
Genus Trentepohlia	0.8	1	0.75	0.857143
Superfamily Tipuloide	0.545455	1	0.545455	0.705882
Genus Monarthropalpus	0.882353	1	0.882353	0.9375
Tribe Lathrobiiini	0.666667	1	0.666667	0.8
Tribe Macrosiphini	1	1	1	1
Genus lachesilla	0	/	0	/
Genus Mycomya	1	1	1	1
Superfamily Culicinae	0.6	0.6	1	0.75
Genus Aedes	0.977273	1	0.977273	0.988506
Genus Rhyssemus	1	1	1	1
Genus Staphylinidae	1	1	1	1
Subfamily Chironominae	1	1	1	1
Genus Rhagio	1	1	1	1

Species Scientific Name	Accuracy	Precision	Recall	F1-Score
Genus Culex	0.8	1	0.8	0.888889
Superfamily Muscoidea	1	1	1	1
Genus Depressaria	1	1	1	1
Superfamily Sciaroidea	1	1	1	1
Genus Molophilus	1	1	1	1
Subtribe Chironomus	1	1	1	1
Family Limoniidae	0.75	1	0.75	0.857143
Genus Mystacides	0.888889	1	0.666667	0.8
Genus Anopheles	0.666667	1	0.666667	0.8
Subgenus Cricotopus	1	/	/	/
Genus Paraproba	0	/	0	/
Clivina Fossor	0	/	0	/
Thaumatomyia notata	0	/	0	/
Psychoda alternata	0	/	0	/
Thaumatomyia notata	0	/	0	/
Family Formicidae	0	0	/	/
Subfamily Araneoidea	0	0	/	/

Appendix D. General AI Pest Identification: Insect Species List and Result

This appendix includes three tables presenting the insect species used to evaluate the AI pest identification tool and their classification results. The sample consists of 1,000 species randomly selected from the most recent U.S. Department of Agriculture (USDA) Regulated Plant Pest Table [22], 262 additional U.S. regulated pest species from other authoritative sources, Pest Tracker [21], and 20 common beneficial insect species [24]. Each species was classified using a standardized ChatGPT prompt to determine whether it is a pest in the U.S., and the responses were collected and analyzed to assess the model's accuracy. The three tables display detailed results for regulated pests, supplementary pests, and beneficial insects.

Table 6. ChatGPT Classification Results for Randomly Sampled USDA-Regulated Pests

Insect Species Name	Classification Result (NO: Non-pests)
Animalia Hemiptera Pentatomidae Ancyrosoma	
Animalia Diptera Cecidomyiidae Contarinia lycopersici	
Animalia Diptera Tephritidae Dyseuaresta gephyrae	
Animalia Hemiptera Blissidae Ischnodemus stalii	
Plantae Salviniales Salviniaceae Salvinia auriculata	
Animalia Coleoptera Buprestidae Chrysobothris capitata	
Animalia Lepidoptera Pyralidae Dioryctria abietella	
Fungi Botryosphaeriales Botryosphaeriaceae Neofusicoccum protearum	
Animalia Tylenchida Criconematidae Hemicriconemoides mangiferae	
Animalia Thysanoptera Thripidae Amalothrips noorazlani	
Animalia Lepidoptera Tortricidae Olethreutes	
Animalia Diptera Chloropidae Eugaurax	
Fungi Helotiales Sclerotiniaceae Monilia numeicola	NO
Plantae Commelinales Commelinaceae Commelina	
Animalia Ixodida Ixodidae Rhipicephalus	
Animalia Lepidoptera Limacodidae Darna pallivitta	
Animalia Lepidoptera Erebidae Eulepidotis juncida	NO

Insect Species Name	Classification Result (NO: Non-pests)
Animalia Stylommatophora Arionidae Arion	
Animalia Coleoptera Chrysomelidae Loxoprosopus	
Fungi Capnodiales Mycosphaerellaceae Pseudocercospora murrayae	
Animalia Thysanoptera Thripidae Frankliniella xanthaner	
Animalia Hemiptera Cicadellidae Zyginella	
Animalia Coleoptera Chrysomelidae Dahlibruchus	
Animalia Coleoptera Scarabaeidae Ancognatha scarabaeoides	
Animalia Diptera Tephritidae Euaresta	
Animalia Thysanoptera Thripidae Thrips rhobdotus	
Animalia Orthoptera Acrididae Melanoplus	
Fungi Xylariales Not Assigned Discula	NO
Animalia Hymenoptera Apidae Xylocopa virginica	NO
Animalia Lepidoptera Tortricidae Argyrotaenia	
Animalia Hymenoptera Cynipidae Iraella ionescui	NO
Animalia Hymenoptera Siricidae Sirex	
Animalia Coleoptera Chrysomelidae Bruchidius uberatus	
Animalia Coleoptera Tenebrionidae Gonocephalum simplex	NO
Animalia Hemiptera Cicadellidae Nephotettix virescens	
Plantae Poales Poaceae Pennisetum pedicellatum	NO
Animalia Lepidoptera Pyralidae Caphys bilineata	
Animalia Hemiptera Diaspididae Semelaspidus artocarpi	
Animalia Collembola Sminthuridae Bourletiella arvalis	NO
Animalia Tylenchida Telotylenchidae Tylenchorhynchus vishwanathensis	
Animalia Hemiptera Cicadellidae Agalliopsis magnifica	
Plantae Fabales Fabaceae Prosopis sericantha	NO

Insect Species Name	Classification Result (NO: Non-pests)
Plantae Lamiales Orobanchaceae Striga asiatica	
Animalia Hemiptera Tingidae Corythucha globigera	
Animalia	
Animalia Coleoptera Curculionidae Endaeus	
Animalia Coleoptera Curculionidae Amphicranus	
Animalia Hemiptera Rhyparochromidae Myodocha unispinosa	
Animalia Hemiptera Lygaeidae Kleidocerys ericae	
Animalia Lepidoptera Erebidae Eudocima tyrannus	
Animalia Hemiptera Dactylopiidae Dactylopius austrinus	
Animalia Thysanoptera Phlaeothripidae	NO
Animalia Lepidoptera Choreutidae Tortyra	NO
Animalia Lepidoptera Lymantriidae	
Animalia Isoptera Termitidae Termes	
Animalia Hemiptera Cicadellidae Erythroneura	
Animalia Hemiptera Coreidae Ceraleptus obtusus	
Animalia Trombidiformes Tenuipalpidae Tenuipalpus	
Animalia Orthoptera Acrididae Acrida	
Plantae Poales Poaceae Urochloa panicoides	NO
Animalia Tylenchida Criconematidae Hemicycliophora chilensis	
Animalia Lepidoptera Pyralidae Homoeosoma	
Animalia Coleoptera Curculionidae Hypoborus	
Animalia Coleoptera Curculionidae Larinus cynarae	
Animalia Orthoptera Gryllidae	NO
Animalia Lepidoptera Gelechiidae Anarsia	
Fungi Not Assigned Not Assigned Plectronidium minor	NO

Insect Species Name	Classification Result (NO: Non-pests)
Fungi Pucciniales Pucciniaceae Puccinia cymbopogonis	
Animalia Lepidoptera Gelechiidae Pectinophora malvella	
Animalia Ixodida Ixodidae Boophilus microplus	
Animalia Coleoptera Chrysomelidae Medythia	
Animalia Stylommatophora Hygromiidae Helicella maritima	NO
Animalia Trombidiformes Tenuipalpidae Brevipalpus chamaedorea	
Animalia Lepidoptera Geometridae Oxydia	
Animalia Coleoptera Curculionidae Ceutorhynchus	
Animalia Hemiptera Thyreocoridae Galgupha	
Animalia Hemiptera Diaspididae Rolaspis	
Animalia Hemiptera Pseudococcidae Dysmicoccus viatorius	
Animalia Hemiptera Pentatomidae Euschistus bifibulus	
Animalia Coleoptera Cerambycidae Ataxia	
Animalia Lepidoptera Erebidae Lymantria xylina	
Animalia Stylommatophora Hygromiidae Xerotricha conspurcata	NO
Animalia Thysanoptera Aeolothripidae Melanthrips	NO
Animalia Hemiptera Pentatomidae Rhaphigaster nebulosa	
Animalia Coleoptera Chrysomelidae Callosobruchus analis	
Fungi Glomerellales Glomerellaceae Colletotrichum kahawae	
Animalia Lepidoptera Crambidae Lineodes	
Plantae Fabales Fabaceae Prosopis torquata	NO
Animalia Hemiptera Lygaeidae Isnodemus	
Animalia Hemiptera Pentatomidae Agonoscelis pubescens	
Animalia Coleoptera Bostrichidae Sinoxylon	
Animalia Coleoptera Curculionidae Faustinus	

Insect Species Name	Classification Result (NO: Non-pests)
Fungi Botryosphaeriales Botryosphaeriaceae Phyllosticta murrayicola	
Plantae Solanales Convolvulaceae Cuscuta chinensis	
Animalia Hemiptera Coccidae Protopulvinaria longivalvata	
Animalia Lepidoptera Noctuidae	
Animalia Coleoptera Curculionidae Exophthalmus scalptus	
Animalia Coleoptera Curculionidae Perperus	
Animalia Stylommatophora Bradybaenidae Pseudobuliminus buliminoides	NO
Animalia Lepidoptera Noctuidae Spodoptera litura	
Animalia Coleoptera Chrysomelidae Amphelasma	
Animalia Coleoptera Bostrichidae Heterobostrychus brunneus	
Animalia Hemiptera Aleyrodidae Rusostigma radiirugosa	
Animalia Hemiptera Cicadellidae Draeculacephala clypeata	
Animalia Hemiptera Membracidae Micrutalis ephippium	NO
Animalia Trombidiformes Eriophyidae Vasates	
Animalia Hemiptera Fulgoridae	
Animalia Hemiptera Coccidae Philephedra	
Animalia Coleoptera Tenebrionidae Epitragus sallaei	NO
Animalia Stylommatophora Enidae Ena	NO
Animalia Hemiptera Tingidae Stephanitis	
Fungi Myriangiales Elsinoaceae Sphaceloma fawcettii scabiosa	
Animalia Coleoptera Cerambycidae Coleoxestia corvina	
Animalia Trombidiformes Tetranychidae Mononychellus tanajoa	
Animalia Coleoptera Curculionidae Stenocarus fuliginosus	
Animalia Hemiptera Aphididae Myzocallis	
Animalia Hymenoptera Formicidae Solenopsis	

**Insect Species Name**	**Classification Result (NO: Non-pests)**
Animalia Trombidiformes Tetranychidae	
Animalia Hemiptera Coccidae Paralecanium	
Animalia Hemiptera Coccidae Trijuba oculata	
Animalia Hemiptera Berytidae Jalysus	NO
Animalia Hemiptera Cicadellidae Acusana	
Animalia Orthoptera Acrididae Stethophyma grossum	
Animalia Hemiptera Pentatomidae Aelia virgata	
Fungi Pleosporales Leptosphaeriaceae Coniothyrium	NO
Animalia Hemiptera Diaspididae Hemiberlesia diffinis	
Animalia Stylommatophora Pleurodontidae Pleurodonte	NO
Animalia Hemiptera Coccidae Cryptinglisia lounsburyi	
Animalia Hemiptera Homotomidae Macrohomotoma gladiata	
Animalia Lepidoptera Erebidae Litoprosopus	
Animalia Orthoptera Acrididae Oedaleus nigrofasciatus	
Animalia Hemiptera Coreidae Scolopocerus	
Fungi Botryosphaeriales Botryosphaeriaceae Phyllosticta micropuncta	
Animalia Lepidoptera Arctiidae Eupantheria alpha	NO
Animalia Stylommatophora Peltellidae Amphibulima	NO
Animalia Coleoptera Curculionidae Ambrosiodmus	
Animalia Eupulmonata Ellobiidae	NO
Fungi Hypocreales Nectriaceae Fusarium oxysporum f. sp. vasinfectum	
Animalia Coleoptera Scarabaeidae Tomarus villosus	
Animalia Coleoptera Curculionidae Xyleborus seriatus	
Fungi Rhytismatales Rhytismataceae Lophodermium petrakii	NO
Animalia Hemiptera Scutelleridae Diolcus	

Insect Species Name	Classification Result (NO: Non-pests)
Fungi Dothideales Planistromellaceae Kellermania	
Animalia Hemiptera Pseudococcidae Vryburgia succulentarum	
Animalia Lepidoptera Lycaenidae Calycopis	NO
Animalia Isoptera Termitidae Nasutitermes corniger	
Animalia Lepidoptera Oecophoridae Agonopterix	NO
Animalia Coleoptera Curculionidae Anacentrinus	
Animalia Lepidoptera Tortricidae Argyrotaenia ljungiana	
Animalia Ixodida Ixodidae Haemaphysalis longicornis	
Animalia Coleoptera Curculionidae Merocnemus	
Animalia Thysanoptera Thripidae Rhipiphorothrips pulchellus	
Animalia Orthoptera Gryllidae Anurogryllus	NO
Animalia Hemiptera Aphalaridae Ctenarytaina peregrina	
Animalia Diptera Anthomyiidae Delia	
Plantae Poales Poaceae Setaria	NO
Animalia Lepidoptera Crambidae Diaphania indica	
Animalia Tylenchida Criconematidae Criconemoides	
Animalia Hemiptera Aleyrodidae Minutaleyrodes minuta	
Animalia Hemiptera Diaspididae Pinnaspis	
Animalia Aphelenchida Aphelenchoididae Aphelenchoides varicaudatus	
Animalia Lepidoptera Erebidae Euclystis	
Animalia Trombidiformes Tetranychidae Tetranychus musae	
Animalia Thysanoptera Thripidae Frankliniella zeteki	
Plantae Fabales Fabaceae Prosopis castellanosii	NO
Animalia Hemiptera Coreidae Archimerus	
Animalia Hemiptera Cerococcidae Cerococcus deklei	

**Insect Species Name**	**Classification Result (NO: Non-pests)**
Animalia Coleoptera Cerambycidae Neocompsa	
Animalia Lepidoptera Alucitidae	NO
Animalia Coleoptera Cerambycidae Semanotus undatus	
Animalia Coleoptera Curculionidae Brachyderes	
Animalia Orthoptera Tettigoniidae Nastonotus foreli	NO
Animalia Hemiptera Coccidae Filippia follicularis	
Animalia Thysanoptera Thripidae Thrips fulvipes	
Animalia Orthoptera Gryllidae Meloimorpha japonicus	NO
Animalia Orthoptera Tettigoniidae Platycleis intermedia	NO
Animalia Coleoptera Cerambycidae Steirastoma marmorata	
Animalia Trombidiformes Tenuipalpidae Brevipalpus chilensis	
Animalia Systellomatophora Veronicellidae Sarasinula plebeia	
Fungi Helotiales Dermateaceae Phloeosporella	NO
Fungi Pucciniales Phakopsoraceae Phakopsora meibomiae	
Fungi Xylariales Amphisphaeriaceae Seimatosporium salicinum	NO
Animalia Hemiptera Eriococcidae Eriococcus lagerstroemiae	
Animalia Hemiptera Psyllidae Psylla	
Animalia Coleoptera Elateridae Aptopus	NO
Animalia Hemiptera Tingidae Corythauma ayyari	
Animalia Hemiptera Pentatomidae Mormidea notulata	
Animalia Hemiptera Cicadellidae Empoasca angustella	
Fungi Pucciniales Phakopsoraceae Phakopsora	
Animalia Lepidoptera Erebidae Eulepidotis rectimargo	NO
Animalia Orthoptera Gryllidae Amphiacusta caraibea	NO
Animalia Lepidoptera Erebidae Apantesis proxima	NO

Insect Species Name	Classification Result (NO: Non-pests)
Fungi Capnodiales Mycosphaerellaceae Pseudocercospora protearum protearum	
Animalia Coleoptera Scarabaeidae Heterochelus	
Animalia Hemiptera Membracidae Vestistilus vacca	NO
Animalia Stylommatophora Ariophantidae Macrochlamys	NO
Animalia Hemiptera Rhyparochromidae Neopamera intermedius	NO
Animalia Coleoptera Tenebrionidae Ulus	NO
Animalia Lepidoptera Erebidae Eudocima	
Animalia Coleoptera Curculionidae Linogeraeus	
Animalia Trombidiformes Tetranychidae Tetranychus macfarlanei	
Animalia Coleoptera Curculionidae Anthonomus melanostictus	
Animalia Orthoptera Romaleidae	NO
Animalia Stylommatophora Philomycidae Meghimatium pictum	NO
Animalia Coleoptera Curculionidae Elytroteinus	
Animalia Coleoptera Bostrichidae Xylobiops	
Animalia Hemiptera Coccidae Ceroplastodes	
Animalia Coleoptera Curculionidae Acanthotomicus	
Animalia Coleoptera Curculionidae Aedemonus erichsoni	
Animalia Hemiptera Diaspididae Andaspis crawii	
Animalia Hemiptera Pentatomidae Eysarcoris modestus	
Animalia Coleoptera Chrysomelidae	
Animalia Hemiptera Aphalaridae Agonoscena	
Animalia Hemiptera Coreidae Coriomeris denticulatus	
Plantae Fabales Fabaceae Mimosa pigra	
Animalia Hemiptera Pachygronthidae Oedancala notata	
Animalia Stylommatophora Hygromiidae Helicella caperata	NO

Insect Species Name	Classification Result (NO: Non-pests)
Animalia Hemiptera Cicadellidae Erythrogonia laudata	
Animalia Hemiptera Lygaeidae Lygaeosoma	
Animalia Thysanoptera Thripidae Thrips major	
Animalia Thysanoptera Thripidae Frankliniella fulvipennis	
Animalia Orthoptera Acrididae Chorthippus	
Animalia Hemiptera Diaspididae Lindingaspis picea	
Animalia Hemiptera Cercopidae Aeneolamia postica	
Fungi Pleosporales Leptosphaeriaceae Coniothyrium septatum	NO
Animalia Lepidoptera Noctuidae Noctua	
Animalia Hemiptera Cydnidae	
Animalia Isoptera Termitidae Gnathamitermes	
Animalia Hemiptera Diaspididae Duplaspidiotus	
Animalia Coleoptera Curculionidae Compsus viridivittatus	
Fungi Pleosporales Phaeosphaeriaceae Stagonospora	
Animalia Hymenoptera Formicidae Crematogaster	
Animalia Lepidoptera Noctuidae Phlogophora meticulosa	
Animalia Hemiptera Coreidae Clavigralla tomentosicollis	
Animalia Thysanoptera Thripidae Frankliniella australis	
Animalia Thysanoptera Thripidae Dichromothrips dendrobii	
Animalia Thysanoptera Thripidae Neohydatothrips samayunkur	
Animalia Hemiptera Pentatomidae Pentatoma rufipes	
Fungi Pucciniales Anamorphic Rust Aecidium	
Animalia Hemiptera Aleyrodidae Dialeurolobus proteae	
Animalia Hemiptera Rhyparochromidae Paragonatas	NO
Animalia Hemiptera Aphididae Sinomegoura citricola	

Insect Species Name	Classification Result (NO: Non-pests)
Animalia Orthoptera Gryllidae Eunemobius	
Animalia Coleoptera Curculionidae Sibinia albovittata	
Animalia Hemiptera Aleyrodidae Paraleyrodes naranjae	
Animalia Coleoptera Curculionidae Coelosternus	
Fungi Pleosporales Didymosphaeriaceae Didymosphaeria	NO
Animalia Hemiptera Tingidae Gargaphia sanchezi	
Animalia Hemiptera Pseudococcidae Maconellicoccus hirsutus	
Animalia Thysanoptera Thripidae Chaetanaphothrips leeuweni	
Animalia Tylenchida Hoplolaimidae Helicotylenchus egyptiensis	
Animalia Hemiptera Cicadellidae Amplicephalus pallidus	
Animalia Hemiptera Aleyrodidae Aleurovitreus	
Animalia Coleoptera Curculionidae Acicnemis	
Animalia Coleoptera Tenebrionidae Nuptis	NO
Animalia Tylenchida Heteroderidae Punctodera	
Animalia Hemiptera Delphacidae Nilaparvata lugens	
Animalia Hemiptera Pseudococcidae Pseudococcus landoi	
Plantae Caryophyllales Amaranthaceae	NO
Animalia Hymenoptera Halictidae Lasioglossum	NO
Fungi Not Assigned Not Assigned Coniothecium	NO
Animalia Orthoptera Acrididae Chortophaga	
Animalia Hemiptera Pyrrhocoridae Pyrrhocoris	NO
Animalia Thysanoptera Thripidae Thrips acaciae	
Animalia Hemiptera Pentatomidae Proxys albopunctulatus	
Animalia Tylenchida Paratylenchidae Paratylenchus obtusicaudatus	
Animalia Orthoptera Acrididae Anacridium rubrispinum	

Insect Species Name	Classification Result (NO: Non-pests)
Animalia Coleoptera Curculionidae Coelositona limosus	
Fungi Botryosphaeriales Botryosphaeriaceae Guignardia citricarpa	
Animalia Diptera Tephritidae Rhagoletis	
Animalia Hemiptera Derbidae Omolicna puertana	
Plantae Poales Poaceae Ischaemum	NO
Animalia Coleoptera Curculionidae Pityokteines curvidens	
Animalia Hemiptera Diaspididae Aspidiotus pacificus	
Animalia Coleoptera Curculionidae Geraeus x-notatum	
Animalia Hemiptera Tingidae Corythaica	
Animalia Hemiptera Coccidae Protopulvinaria	
Animalia Coleoptera Cerambycidae	
Animalia Coleoptera Apionidae Trichapion pauper	
Animalia Hemiptera Rhyparochromidae Heraeus itzelae	NO
Animalia Phasmida Phasmatidae Aplopus	NO
Fungi Amphisphaeriales Pestalotiopsidaceae Pestalotiopsis torrendii	
Animalia Hemiptera Miridae Dagbertus	
Animalia Hemiptera Membracidae Aconophora mexicana	
Animalia Coleoptera Chrysomelidae Oulema	
Animalia Hymenoptera Argidae Sphacophilus	NO
Fungi Pleosporales Not Assigned Phoma thujana	
Fungi Botryosphaeriales Botryosphaeriaceae Diplodia palmicola	
Animalia Hemiptera Rhyparochromidae Botocudo	NO
Animalia Thysanoptera Thripidae Frankliniella phaeaner	
Animalia Hemiptera Heterogastridae	NO
Animalia Orthoptera Tettigoniidae Caulopsis	NO

Insect Species Name	Classification Result (NO: Non-pests)
Animalia Stylommatophora Arionidae Arion Arvc	
Animalia Coleoptera Chrysomelidae Distigmoptera	
Animalia Coleoptera Curculionidae Anchonus	
Animalia Coleoptera Scarabaeidae Epectinaspis	
Animalia Hemiptera Coccidae Pulvinaria tenuivalvata	
Animalia Coleoptera Cerambycidae Oemona hirta	
Animalia Lepidoptera Saturniidae Hylesia	
Animalia Coleoptera Elateridae Glyphonyx	
Animalia Hemiptera Coreidae Leptoglossus chiliensis	
Animalia Coleoptera Cerambycidae Euryscelis suturalis	
Animalia Lepidoptera Crambidae Duponchelia	
Animalia Lepidoptera Noctuidae Elaphria subrubens	
Animalia Hemiptera Psyllidae Euceropsylla	
Animalia Hemiptera Membracidae Trichaetipyga infantilis	
Animalia Lepidoptera Tortricidae	
Animalia Coleoptera Curculionidae Myossita	
Animalia Hymenoptera Siricidae Urocerus gigas	
Animalia Hymenoptera Siricidae Sirex noctilio	
Fungi Pucciniales Pucciniaceae Uromyces alyxiae	
Animalia Coleoptera Curculionidae Sternocoelus	
Animalia Coleoptera Curculionidae Diaprepes abbreviatus	
Animalia Coleoptera Curculionidae Desiantha diversipes	
Fungi Glomerellales Glomerellaceae Colletotrichum	
Animalia Diptera Anthomyiidae Hylemya	
Animalia Lepidoptera Noctuidae Phlogophora	

**Insect Species Name**	**Classification Result (NO: Non-pests)**
Animalia Hemiptera Rhyparochromidae Paracholula	NO
Animalia Hemiptera Cicadellidae Erythridula noeva	
Animalia Hemiptera Lygaeidae Arocatus longiceps	
Animalia Hemiptera Aleyrodidae Metaleurodicus griseus	
Animalia Hemiptera Tingidae Corythucha fuscomaculata	
Fungi Capnodiales Mycosphaerellaceae Asperisporium	
Animalia Thysanoptera Thripidae Thrips annulatus	
Animalia Stylommatophora Helicidae Otala	NO
Animalia Hemiptera Cicadellidae Eupteryx	
Fungi Helotiales Dermateaceae Marssonina	
Animalia Trombidiformes Eriophyidae Aceria litchii	
Animalia Coleoptera Buprestidae Chalcophora	
Animalia Stylommatophora Pleurodontidae Pleurodonte guadeloupensis dominicana	NO
Animalia Coleoptera Bostrichidae Stephanopachys	
Animalia Hemiptera Diaspididae Aulacaspis bambusae	
Animalia Coleoptera Chrysomelidae Cassida circumdata	
Animalia Lepidoptera Pterophoridae Stenoptilia	NO
Animalia Hemiptera Pentatomidae Brachynema germarii	
Animalia Lepidoptera Gelechiidae	
Animalia Coleoptera Buprestidae Chrysobothris	
Plantae Myrtales Myrtaceae Melaleuca quinquenervia	
Animalia Coleoptera Dryophthoridae Acantharhinus dregei	
Animalia Hemiptera Lygaeidae Xyonysius	
Animalia Orthoptera Acrididae Hieroglyphus	
Animalia Lepidoptera Tortricidae Strepsicrates	

Insect Species Name	Classification Result (NO: Non-pests)
Animalia Coleoptera Curculionidae Coccotrypes	
Animalia Lepidoptera Erebidae Cosmosoma	NO
Animalia Hemiptera Cydnidae Macroscytus brunneus	
Animalia Diptera Tephritidae Bactrocera	
Animalia Hemiptera Pseudococcidae Phenacoccus stelli	
Animalia Hemiptera Pseudococcidae Pseudococcus philippinicus	
Animalia Hymenoptera Formicidae Pheidole	
Animalia Trombidiformes Tarsonemidae Tarsonemus hermes	
Animalia Hemiptera Pentatomidae Chroantha ornatula	
Animalia Orthoptera Pyrgomorphidae Atractomorpha	
Animalia Coleoptera Chrysomelidae Calligrapha	
Animalia Coleoptera Chrysomelidae Odontota	
Fungi Diaporthales Diaporthaceae Phomopsis juniperivora	
Fungi Erysiphales Erysiphaceae Oidium	
Animalia Hemiptera Coccidae Neoplatylecanium	
Animalia Coleoptera Curculionidae Hylesinus	
Animalia Coleoptera Buprestidae Agrilus biguttatus	
Animalia Hemiptera Pentatomidae Mecidea	
Animalia Hemiptera Coccidae Udinia catori	
Animalia Diptera Muscidae	
Animalia Trombidiformes Tarsonemidae Steneotarsonemus	
Animalia Coleoptera Scarabaeidae Oxythyrea funesta	
Animalia Systellomatophora Veronicellidae Colosius confusus	NO
Animalia Hemiptera Miridae Orthops campestris	
Animalia Trombidiformes Tetranychidae Tetranychus lambi	

**Insect Species Name**	**Classification Result (NO: Non-pests)**
Animalia Hemiptera Diaspididae Furcaspis scleroprymna	
Animalia Stylommatophora Peltellidae Amphibulima patula	NO
Animalia Coleoptera Cerambycidae Clytus rhamni	
Animalia Hemiptera Aphididae Dysaphis cynarae	
Plantae Alismatales Alismataceae	NO
Animalia Lepidoptera Nymphalidae Aglais urticae	NO
Bacteria Xanthomonadales Xanthomonadaceae Xanthomonas (cancrosis b)	
Plantae Solanales Solanaceae Solanum viarum	
Fungi Microascales Microascaceae Scopulariopsis	NO
Animalia Tylenchida Rotylenchulidae Rotylenchulus macrodoratus	
Animalia Coleoptera Curculionidae Smicronyx	
Animalia Coleoptera Chrysomelidae Strabala	
Animalia Hemiptera Oxycarenidae Macroplax fasciata	
Animalia Lepidoptera Pyralidae	
Animalia Lepidoptera Tortricidae Sparganothis	
Animalia Stylommatophora Bradybaenidae Fruticicola fruticum	NO
Animalia Hemiptera Pentatomidae Stenozygum coloratum	
Animalia Stylommatophora Hygromiidae Cernuella cf. virgata	NO
Animalia Coleoptera Tenebrionidae Caedius	NO
Animalia Hymenoptera Diprionidae	
Animalia Coleoptera Curculionidae Scolytoplatypus	
Fungi Pleosporales Pleosporaceae Teratosphaeria bellula	
Animalia Coleoptera Chrysomelidae Chelymorpha pubescens	
Animalia Coleoptera Curculionidae Chalcodermus angulicollis	
Fungi Pleosporales Tubeufiaceae Acanthophiobolus	NO

**Insect Species Name**	**Classification Result (NO: Non-pests)**
Animalia Coleoptera Curculionidae Misophrice	
Animalia Hemiptera Aleyrodidae Paraleyrodes pseudonaranjae	
Fungi Pleosporales Pleosporaceae Bipolaris musae-sapientium	
Animalia Lepidoptera Noctuidae Trichoplusia spoliata	
Animalia Hemiptera Lygaeidae Ochrostomus bilimeki	
Fungi Helotiales Sclerotiniaceae Botrytis	
Animalia Hemiptera Rhyparochromidae Elasmolomus pallens	
Animalia Coleoptera Cerambycidae Ropica	
Animalia Coleoptera Bostrichidae Calopertha truncatula	
Animalia Lepidoptera Erebidae Empyreuma anassa	NO
Animalia Hemiptera Pseudococcidae Pseudococcus zamiae	
Chromista Peronosporales Pythiaceae Pythium	
Animalia Hemiptera Acanaloniidae	
Animalia Hemiptera Membracidae Aconophora	
Animalia Coleoptera Curculionidae Bothinodontes	
Animalia Hemiptera Rhyparochromidae Raglius confusus	NO
Animalia Hemiptera Cydnidae Adomerus	
Animalia Coleoptera Brentidae Cylas punctipennis	
Animalia Coleoptera Bostrichidae Minthea obsita	
Animalia Hemiptera Cicadellidae Homalodisca vitripennis	
Animalia Hemiptera Aleyrodidae Aleuroclava euryae	
Animalia Lepidoptera Erebidae Cosmosoma auge	NO
Animalia Orthoptera Acrididae Schistocerca	
Animalia Lepidoptera Noctuidae Pseudaletia impuncta	
Animalia Coleoptera Cerambycidae Batocera	

**Insect Species Name**	**Classification Result (NO: Non-pests)**
Animalia Hemiptera Aleyrodidae Aleuroclava gordoniae	
Animalia Hemiptera Halimococcidae Colobopyga	
Animalia Coleoptera Chrysomelidae Euphrytus	
Animalia Diptera Tephritidae Myiopardalis	
Fungi Pleosporales Not Assigned Phoma	
Animalia Coleoptera Cerambycidae Lagocheirus simplicicornis	
Animalia Stylommatophora Hygromiidae Xeropicta candaharica	NO
Fungi Albuginales Albuginaceae Pustula centaurii	
Animalia Lepidoptera Saturniidae Saturnia	NO
Animalia Hymenoptera Tenthredinidae Nematus	
Animalia Hemiptera Miridae Closterotomus	
Animalia Hemiptera Cicadellidae Acinopterus	
Animalia Coleoptera Scarabaeidae Phyllophaga	
Animalia Tylenchida Hoplolaimidae Scutellonema magniphasma	
Animalia Coleoptera Scarabaeidae Sericoides	
Animalia Hemiptera Coreidae Amblypelta cocophaga	
Animalia Coleoptera Cerambycidae Malobidion grande	
Animalia Hemiptera Pseudococcidae Palmicultor	
Animalia Lepidoptera Tortricidae Cydia splendana	
Fungi Ustilaginales Ustilaginaceae Sporisorium	
Animalia Coleoptera Curculionidae Cactophagus graphipterus	
Fungi Pleosporales Not Assigned Pyrenochaeta	
Animalia Hemiptera Cicadellidae Eupelix cuspidata	
Plantae Santalales Loranthaceae Dendropemon	NO
Animalia Lepidoptera Cosmopterigidae Cosmopterix	NO

Insect Species Name	Classification Result (NO: Non-pests)
Animalia Stylommatophora Succineidae Succinea rubella	NO
Animalia Coleoptera Curculionidae Sophrorhinus	
Animalia Hymenoptera Eurytomidae Bephratelloides pomorum	
Animalia Coleoptera Cerambycidae Trachyderes succinctus succinctus	
Fungi Tilletiales Tilletiaceae Neovossia	
Animalia Hemiptera Pseudococcidae Paracoccus cavaticus	
Animalia Lepidoptera Gelechiidae Schneidereria pistaciicola	
Animalia Hemiptera Aleyrodidae Metaleurodicus minimus	
Animalia Hemiptera Membracidae Aconophora compressa	
Animalia Lepidoptera Tortricidae Cryptophlebia leucotreta	
Plantae Salviniales Salviniaceae Salvinia molesta	
Animalia Lepidoptera Tortricidae	
Animalia Coleoptera Erirhinidae Onychylis setiger	
Animalia Thysanoptera Thripidae Rhipiphorothrips	
Animalia Hemiptera Diaspididae Lepidosaphes pistaciae	
Plantae Lamiales Acanthaceae Hygrophila polysperma	
Animalia Lepidoptera Erebidae Eucereon metoidesis	NO
Animalia Isoptera Rhinotermitidae Heterotermes tenuis	
Animalia Coleoptera Chrysomelidae Acanthoscelides guazumae	
Animalia Hemiptera Cicadellidae Allygidius abbreviatus	
Animalia Lepidoptera Crambidae Diatraea lineolata	
Animalia Stylommatophora Subulinidae Leptinaria unilamellata	NO
Animalia Lepidoptera Hesperiidae Epargyreus	NO
Animalia Hemiptera Tingidae Dictyla	
Animalia Hemiptera Diaspididae Pinnaspis uniloba	

Insect Species Name	Classification Result (NO: Non-pests)
Animalia Hemiptera Clastopteridae Clastoptera biguttata	
Animalia Lepidoptera Tortricidae Epiphyas	
Animalia Coleoptera Curculionidae Hylurgops	
Animalia Dorylaimida Longidoridae Xiphinema incognitum	
Animalia Tylenchida Pratylenchidae Pratylenchus loosi	
Animalia Hemiptera Aphididae	
Animalia Hemiptera Miridae Rhinacloa mysteriosus	
Animalia Lepidoptera Erebidae Lymantria dispar	
Animalia Orthoptera Tettigoniidae Ancistrocercus circumdatus	NO
Animalia Coleoptera Scarabaeidae Athlia rustica	
Animalia Hemiptera Scutelleridae Camirus	
Animalia Lepidoptera Depressariidae	NO
Animalia Coleoptera Bostrichidae Stephanopachys quadricollis	
Plantae Polygonales Polygonaceae Emex australis	
Animalia Hemiptera Tessaratomidae Tessaratoma	
Animalia Coleoptera Curculionidae Metriophilus	
Animalia Hemiptera Lygaeidae Arocatus melanocephalus	
Animalia Lepidoptera Noctuidae Leucania putrescens	
Animalia Orthoptera Mogoplistidae Hoplosphyrum	NO
Fungi Xylariales Amphisphaeriaceae Truncatella	
Animalia Hemiptera Pentatomidae Sciocoris maculatus	
Animalia Thysanoptera Thripidae Pseudanaphothrips achaetus	
Animalia Lepidoptera Pyralidae	
Animalia Lepidoptera Doidae	NO
Animalia Lepidoptera Incurvariidae	NO

Insect Species Name	Classification Result (NO: Non-pests)
Animalia Diptera Agromyzidae Agromyza	
Animalia Coleoptera Chrysomelidae Caryedes brasiliensis	
Fungi Pucciniales Phragmidiaceae Phragmidium	
Animalia Coleoptera Elateridae Conoderus semimarginatus	
Animalia Coleoptera Cerambycidae Mecometopus	
Animalia Hemiptera Cicadellidae Macrosteles	
Animalia Coleoptera Chrysomelidae Meibomeus	
Fungi Capnodiales Mycosphaerellaceae Pseudocercosporella	
Animalia Hemiptera Aleyrodidae Aleuroclava	
Animalia Hemiptera	NO
Animalia Orthoptera Mogoplistidae	NO
Animalia Lepidoptera Crambidae Chilo suppressalis	
Animalia Tylenchida Criconematidae	
Animalia Trombidiformes Tarsonemidae Neotarsonemoides occultus	
Animalia Hemiptera Cicadellidae Osbornellus ecuadoricus	
Animalia Coleoptera Chrysomelidae Anisostena	
Animalia Coleoptera Cerambycidae	
Animalia Dorylaimida Longidoridae Xiphinema incertum	
Animalia Isoptera Termitidae Anoplotermes	NO
Animalia Coleoptera Chrysomelidae Nisotra	
Animalia Trombidiformes Tarsonemidae Excelsotarsonemus	
Fungi Not Assigned Not Assigned Camarosporula persoonii	
Animalia Orthoptera Gryllidae Trigonidomimus annuliger	NO
Animalia Lepidoptera Notodontidae Thaumetopoea processionea	
Animalia Stylommatophora Helicidae Pseudotachea splendida	NO

**Insect Species Name**	**Classification Result (NO: Non-pests)**
Animalia Coleoptera Chrysomelidae Mimosestes	
Animalia Hemiptera Aphididae Macrosiphoniella	
Animalia Coleoptera Erirhinidae Tadius	
Animalia Coleoptera Scarabaeidae Hylamorpha elegans	
Animalia Diptera Tephritidae Toxotrypana curvicauda	
Viruses Not Assigned Geminiviridae Begomovirus ageratum enation virus (AEV)	
Animalia Orthoptera Pyrgomorphidae Sphenarium purpurascens	
Animalia Coleoptera Chrysomelidae Diabrotica undecimpunctata duodecimnotata	
Animalia Coleoptera Elateridae Ectinus aterrimus	
Animalia Lepidoptera Noctuidae Spodoptera littoralis	
Animalia Diptera Tephritidae Ceratitis	
Animalia Coleoptera Chrysomelidae Heikertingerella	
Animalia Coleoptera Tenebrionidae Blapstinus tibialis	
Animalia Hemiptera Blissidae Ischnodemus quadratus	
Animalia Thysanoptera Thripidae Dorcadothrips	
Animalia Coleoptera Curculionidae Pandeleteius	
Animalia Hemiptera Cydnidae Macroscytus	
Animalia Orthoptera Tettigoniidae Conocephalus saltator	NO
Bacteria Xanthomonadales Xanthomonadaceae Xanthomonas axonopodis pv. vasculorum	
Animalia Stylommatophora Hygromiidae Cernuella	NO
Animalia Hymenoptera Megachilidae Anthidium manicatum	
Animalia Hemiptera Scutelleridae Chrysocoris stollii	
Animalia Hemiptera Pentatomidae Sciocoris conspurcatus	
Animalia Hemiptera Aleyrodidae Aleurotuberculat aucubae	
Animalia Hemiptera Rhyparochromidae Ozophora concava	

Insect Species Name	Classification Result (NO: Non-pests)
Animalia Hemiptera Rhyparochromidae Sphragisticus	
Animalia Hemiptera Aetalionidae Aetalion	
Animalia Coleoptera Chrysomelidae Ceroclytra	
Animalia Lepidoptera Erebidae Catocala	NO
Animalia Hemiptera Diaspididae Lepidosaphes cornuta	
Animalia Hemiptera Rhopalidae Arhyssus peruensis	
Plantae Santalales Viscaceae	NO
Animalia Stylommatophora Hygromiidae Trochoidea elegans	NO
Animalia Hemiptera Cixiidae Bothriocera	
Animalia Coleoptera Scarabaeidae Diplotaxis	
Animalia Hemiptera Miridae Apolygus	
Animalia Coleoptera Scarabaeidae Serica	
Animalia Hemiptera Rhyparochromidae Rhyparochromus adspersus	
Animalia Hemiptera Rhyparochromidae Cligenes	
Animalia Orthoptera Acrididae	
Animalia Diptera Syrphidae	NO
Animalia Hemiptera Cicadellidae Graphocephala simulata	
Animalia Hemiptera Pseudococcidae Dysmicoccus texensis	
Fungi Glomerellales Glomerellaceae Glomerella	
Animalia Hemiptera Coreidae Gonocerus acuteangulatus	
Animalia Hemiptera Blissidae Ischnodemus variegatus	
Animalia Stylommatophora Megalobulimidae Megalobulimus oblongus	NO
Animalia Hemiptera Miridae Pachymerocorista pilosus	
Animalia Hemiptera Cicadellidae Arboridia parvula	
Animalia Coleoptera Curculionidae Hypothenemus	

Insect Species Name	Classification Result (NO: Non-pests)
Animalia Coleoptera Curculionidae Renocis	
Animalia Hemiptera Diaspididae Pseudoparlatoria carolilehmanni	
Animalia Coleoptera Tenebrionidae Opatroides punctulatus	
Animalia Hemiptera Diaspididae Andaspis	
Animalia Lepidoptera Psychidae	NO
Animalia Phasmatodea Heteronemiidae Carausius morosus	NO
Animalia Hemiptera Pentatomidae Glaucias subpunctatus	
Animalia Coleoptera Chrysomelidae Charidotella trisignata	
Animalia Thysanoptera Thripidae Neohydatothrips variabilis	
Animalia Coleoptera Chrysomelidae Bruchidius atrolineatus	
Animalia Coleoptera Chrysomelidae Zabrotes	
Animalia Coleoptera Chrysomelidae Brontispa longissima	
Animalia Isoptera Kalotermitidae Glyptotermes	
Animalia Thysanoptera Thripidae Astrothrips	
Fungi Helotiales Dermateaceae Cryptosporiopsis	
Animalia Coleoptera Scarabaeidae Euphoria lurida	
Animalia Lepidoptera Erebidae Virbia rosenbergi	NO
Plantae Santalales Loranthaceae Taxillus	NO
Animalia Hemiptera Coreidae Anasa uhleri	
Animalia Hemiptera Pentatomidae Halyomorpha	
Animalia Coleoptera Curculionidae Heilipus lauri	
Animalia Coleoptera Cerambycidae Dectes	
Animalia Hemiptera Margarodidae Icerya	
Animalia Lepidoptera Arctiidae Amsacta morei	NO
Animalia Hemiptera Rhyparochromidae Brentiscerus	

**Insect Species Name**	**Classification Result (NO: Non-pests)**
Animalia Lepidoptera Oecophoridae Stathmopoda	NO
Animalia Hemiptera Aleyrodidae Aleuroclava latus	
Animalia Stylommatophora Hygromiidae Xeropicta	NO
Animalia Coleoptera Chrysomelidae Meibomeus apicicornis	
Animalia Trombidiformes Eriophyidae Cecidophyopsis ribis	
Animalia Coleoptera Buprestidae Belionota	
Animalia Coleoptera Tenebrionidae Lyprops	
Animalia Hemiptera Cicadellidae Ceratagallia	
Animalia Lepidoptera	NO
Animalia Hemiptera Aleyrodidae Aleuroclava magnoliae	
Animalia Hemiptera Pentatomidae Podops inuncta	
Animalia Diptera Anthomyiidae	NO
Animalia Coleoptera Tenebrionidae Lobometopon	
Fungi Pleosporales Leptosphaeriaceae Leptosphaeria leucadendri	
Animalia Hemiptera Diaspididae Dynaspidiotus abieticola	
Animalia Tylenchida Hoplolaimidae Hoplolaimus	
Animalia Coleoptera Buprestidae Phaenops	
Animalia Hemiptera Pseudococcidae Pseudantonina	
Animalia Lepidoptera Crambidae Herpetogramma	
Plantae Lamiales Plantaginaceae Limnophila	NO
Animalia Coleoptera Brentidae Coptorrhynchus	
Animalia Hemiptera Diaspididae Unaspis yanonensis	
Animalia Trombidiformes Tetranychidae Tetranychus (Polynychus)	
Animalia Hemiptera Aclerdidae Aclerda takahashii	
Animalia Hymenoptera Tenthredinidae Dolerus	

Insect Species Name	Classification Result (NO: Non-pests)
Animalia Lepidoptera Oecophoridae Cerconota	NO
Animalia Coleoptera Erirhinidae Neohydronomus elegans	
Animalia Dorylaimida Longidoridae Xiphinema basiri	
Animalia Coleoptera Curculionidae Pityophthorus	
Animalia Stylommatophora Arionidae Arion subfuscus	NO
Animalia Hemiptera Miridae	NO
Fungi Glomerellales Glomerellaceae Colletotrichum (long-spored)	
Animalia Hemiptera Aetalionidae Aetalion reticulatum	
Animalia Isoptera Rhinotermitidae Coptotermes havilandi	
Animalia Coleoptera Chrysomelidae Epitrix pubescens	
Animalia Stylommatophora Hygromiidae Cernuella cf. neglecta	NO
Animalia Orthoptera Gryllidae Acheta hispanicus	NO
Fungi Not Assigned Plectosphaerellaceae Verticillium	
Animalia Coleoptera Curculionidae Pityogenes bidentatus	
Animalia Coleoptera Buprestidae Taphrocerus	
Fungi Pleosporales Not Assigned Phoma tracheiphila	
Animalia Lepidoptera Carposinidae Carposina	
Animalia Coleoptera Curculionidae Exophthalmus quadrivittatus	
Animalia Trombidiformes Tetranychidae Oligonychus annonicus	
Animalia Hemiptera Miridae Pachytomella passerinii	
Animalia Coleoptera Cerambycidae Oreodera corticina	
Animalia Hemiptera Diaspididae Neoleonardia alata	
Animalia Coleoptera Brentidae	
Animalia Hemiptera Pseudococcidae Formicococcus njalensis	
Animalia Coleoptera Chrysomelidae Isotes mexicana	

Insect Species Name	Classification Result (NO: Non-pests)
Animalia Coleoptera Bostrichidae Sinoxylon crassum	
Animalia Hemiptera Pentatomidae Bagrada	
Animalia Coleoptera Cerambycidae Cerambyx	
Animalia Tylenchida Telotylenchidae	
Animalia Coleoptera Curculionidae Orsophagus	
Animalia Orthoptera Acrididae Brachycryotaphus	
Animalia Coleoptera Curculionidae Rhyssomatus strangulatus	
Animalia Hemiptera Diaspididae Parlatoria	
Animalia Hemiptera Pentatomidae Glaucias	
Animalia Coleoptera Cerambycidae Compsibidion vanum	
Animalia Hemiptera Pseudococcidae Balanococcus takahashii	
Animalia Coleoptera Chrysomelidae Phenrica	
Animalia Coleoptera Curculionidae Scolytus mundus	
Animalia Systellomatophora Veronicellidae Semperula siamensis	NO
Animalia Stylommatophora Helicarionidae Elisolimax flavescens	NO
Animalia Hemiptera Aleyrodidae Crenidorsum	
Fungi Pucciniales Phakopsoraceae Crossopsora ziziphi	
Animalia Lepidoptera Pyralidae Genopaschia	
Animalia Hemiptera Cicadellidae Agallia constricta cubana	
Animalia Hemiptera Ortheziidae	
Fungi Capnodiales Mycosphaerellaceae Mycosphaerella pandani	
Fungi Botryosphaeriales Botryosphaeriaceae Phyllosticta	
Animalia Hemiptera Diaspididae Aonidiella	
Animalia Coleoptera Curculionidae Faustinus rhombifer	
Animalia Trombidiformes Tetranychidae Oligonychus psidium	

Insect Species Name	Classification Result (NO: Non-pests)
Animalia Hymenoptera Torymidae	
Animalia Thysanoptera Phlaeothripidae Haplothrips clarisetis	
Animalia Coleoptera Curculionidae Platypus	
Animalia Hemiptera Diaspididae Leonardianna	
Animalia Hemiptera Asterolecaniidae Palmaspis inlabefacta	
Animalia Hemiptera Rhopalidae Harmostes gravidator	
Animalia Trombidiformes Tetranychidae Bryobia repensi	
Animalia Lepidoptera Noctuidae Bleptina	
Fungi Botryosphaeriales Botryosphaeriaceae Botryosphaeria lutea	
Animalia Lepidoptera Pyralidae	NO
Animalia Coleoptera Curculionidae Palaeopus	
Animalia Coleoptera Cerambycidae Psapharochrus circumflexus	
Animalia Triplonchida Trichodoridae Trichodorus viruliferus	
Fungi Capnodiales Mycosphaerellaceae Pseudocercospora myrticola	
Fungi Glomerellales Glomerellaceae Colletotrichum queenslandicum	
Animalia Coleoptera Curculionidae Tomicus destruens	
Animalia Hemiptera Pseudococcidae Paracoccus herreni	
Animalia Lepidoptera Oecophoridae Ethmia	NO
Animalia Thysanoptera Thripidae Thrips alliorum	
Animalia Coleoptera Chrysomelidae Neolochmaea	
Animalia Hemiptera Cicadellidae Ollarianus	
Animalia Coleoptera Chrysomelidae Allochroma fasciatum	
Animalia Diptera Tephritidae Dacus cucurbitae	
Animalia Hemiptera Pentatomidae Tibraca limbativentris	
Animalia Coleoptera Chrysomelidae Acanthoscelides obreptus	

**Insect Species Name**	**Classification Result (NO: Non-pests)**
Animalia Orthoptera Acrididae Chorthippus albomarginatus	NO
Fungi Pucciniales Chaconiaceae Hemileia vastatrix	
Animalia Tylenchida Telotylenchidae Tylenchorhynchus goffarti	
Animalia Lepidoptera Erebidae Ceramidia	NO
Animalia Systellomatophora Veronicellidae Pseudoveronicella	NO
Animalia Hemiptera Alydidae Leptocorisa	
Animalia Stylommatophora Succineidae Indosuccinea	NO
Animalia Coleoptera Chrysomelidae Mimosestes humeralis	
Animalia Hemiptera Coccidae Pulvinaria ixorae	
Animalia Hemiptera Delphacidae Asiraca clavicornis	
Animalia Hemiptera Psyllidae Cacopsylla pyricola	
Animalia Lepidoptera Tineidae Nemapogon gerasimovi	NO
Animalia Trombidiformes Tarsonemidae Tarsonemus lobosus	
Animalia Hemiptera Cicadellidae Mexigina	
Animalia Hemiptera Membracidae Ennya	
Animalia Stylommatophora Subulinidae Subulina	NO
Fungi Not Assigned Not Assigned Monodictys	
Fungi Pleosporales Phaeosphaeriaceae Phaeosphaeria palmarum	
Animalia Hemiptera Membracidae Cladonota	
Animalia Coleoptera Curculionidae Pandeleteinus magdalenensis	
Fungi Phyllachorales Phaeochoraceae Phaeochora steinheilii	
Fungi Capnodiales Mycosphaerellaceae Pseudocercospora eriobotryicola	
Fungi Pucciniales Coleosporiaceae Chrysomyxa ledi	
Animalia Lepidoptera Erebidae Pareuchaetes	
Animalia Hemiptera Rhyparochromidae Eremocoris fenestratus	

Insect Species Name	Classification Result (NO: Non-pests)
Animalia Lepidoptera Crambidae Conogethes punctiferalis	
Animalia Coleoptera Curculionidae Naupactus xanthographus	
Animalia Hymenoptera Andrenidae	NO
Animalia Hemiptera Lygaeidae Graptostethus	
Animalia Coleoptera Scarabaeidae Epectinaspis moreletiana	
Animalia Hymenoptera Tenthredinidae	NO
Animalia Hemiptera Cicadellidae Alconeura	
Animalia Hemiptera Coccidae Cryptinglisia ziziphi	
Animalia Coleoptera Chrysomelidae Calligrapha elegantula	
Animalia Stylommatophora Helicarionidae Ovachlamys fulgens	NO
Animalia Hemiptera Aphididae Periphyllus	
Animalia Coleoptera Cerambycidae Psapharochrus lateralis	
Animalia Hemiptera Delphacidae Javesella pellucida	
Animalia Coleoptera Scarabaeidae Strigoderma	
Animalia Orthoptera Acrididae Acrida cinerea	NO
Animalia Hemiptera Alydidae Hyalymenus	
Animalia Coleoptera Cerambycidae Xylotrechus hircus	
Animalia Coleoptera Chrysomelidae Charidotella bicolor	
Animalia Coleoptera Curculionidae Dactylotrypes longicollis	
Animalia Hemiptera Miridae Lygocoris	
Animalia Lepidoptera Crambidae Parapoynx stagnalis	
Fungi Capnodiales Mycosphaerellaceae Cercospora corchori	
Animalia Coleoptera Bostrichidae Heterobostrychus hamatipennis	
Animalia Hemiptera Cydnidae Sehirus	
Animalia Lepidoptera Pieridae Catopsilia pomona	

Insect Species Name	Classification Result (NO: Non-pests)
Animalia Coleoptera Chrysomelidae Pachymerus	
Animalia Hemiptera Pseudococcidae Nipaecoccus annonae	
Animalia Diptera Agromyzidae Ophiomyia phaseoli	
Animalia Hemiptera Aleyrodidae Aleurodicus ornatus	
Animalia Coleoptera Elateridae Deromecus	
Animalia Lepidoptera Gracillariidae Caloptilia	
Animalia Hemiptera Scutelleridae Phimodera	
Animalia Hemiptera Cicadellidae Cicadulina tortilla	
Animalia Coleoptera Scarabaeidae Apogonia	
Animalia Coleoptera Tenebrionidae	
Animalia Coleoptera Cerambycidae Plagionotus arcuatus	
Animalia Coleoptera Bostrichidae Sinoxylon anale	
Animalia Hemiptera Aclerdidae Aclerda	
Animalia Hemiptera Cydnidae Amnestus uhleri	
Animalia Lepidoptera Sesiidae Synanthedon	
Animalia Lepidoptera Tortricidae Cydia araucariae	
Animalia Coleoptera Dryophthoridae Mesocordylus scutellaris	
Animalia Coleoptera Chrysomelidae Chrysomela mexicana	
Fungi Botryosphaeriales Botryosphaeriaceae Sphaeropsis	
Animalia Orthoptera Acrididae	
Animalia Lepidoptera Erebidae Metria euristea	
Animalia Coleoptera Scarabaeidae Adoretus sinicus	
Animalia Hemiptera Aleyrodidae Aleuroglandulus inanis	
Animalia Coleoptera Chrysomelidae Metrioidea	
Animalia Lepidoptera Noctuidae Thysanoplusia orichalcea	

**Insect Species Name**	**Classification Result (NO: Non-pests)**
Animalia Lepidoptera Tortricidae Gymnandrosoma aurantianum	
Animalia Lepidoptera Gelechiidae Tuta absoluta	
Animalia Hemiptera Alydidae Riptortus	
Animalia Hemiptera Pseudococcidae Fonscolombia europaea	
Animalia Coleoptera Curculionidae Sibinia arenariae	
Animalia Hemiptera Miridae Pycnoderes	
Animalia Stylommatophora Arionidae Arion ater	NO
Animalia Tylenchida Criconematidae Criconema duplicivestitum	
Animalia Coleoptera Apionidae Trichapion	
Animalia Coleoptera Curculionidae Conotrachelus perseae	
Animalia Hemiptera Cicadellidae Dechacona missionum	
Animalia Hemiptera Lygaeidae Melanocoryphus	
Fungi Capnodiales Mycosphaerellaceae Cercospora	
Animalia Hemiptera Rhyparochromidae	
Animalia Hemiptera Pseudococcidae Kermicus	
Animalia Coleoptera Elateridae Conoderus sinaloae	
Animalia Thysanoptera Thripidae Tusothrips	
Fungi Capnodiales Teratosphaeriaceae Batcheloromyces	
Animalia Hemiptera Miridae Cyrtocapsus femoralis	
Animalia Lepidoptera Crambidae Maruca vitrata	
Animalia Hemiptera Tingidae Macrotingis novicis	
Animalia Hemiptera Lygaeidae Lygaeinae	
Animalia Hemiptera Flatidae Poekilloptera phalaenoides	
Fungi Pucciniales Anamorphic Rust Aecidium evansii	
Animalia Orthoptera Gryllidae Microgryllus	NO

Insect Species Name	Classification Result (NO: Non-pests)
Animalia Hemiptera Pentatomidae Rhyssocephala	
Animalia Hemiptera Plataspidae Coptosoma cribraria	
Animalia Orthoptera Acrididae Calliptamus barbarus	NO
Animalia Stylommatophora Helicidae Marmorana serpentina	NO
Animalia Coleoptera Curculionidae Plocetes sickingiae	
Animalia Hemiptera Cicadellidae Osbornellus infuscatus	
Animalia Lepidoptera Tortricidae	NO
Animalia Thysanoptera Thripidae Danothrips trifasciatus	
Animalia Coleoptera Curculionidae Diocalandra taitensis	
Animalia Hemiptera Lygaeidae Polychisme ferruginosus	
Animalia Lepidoptera Noctuidae Earias	
Animalia Hemiptera Alydidae Camptopus lateralis	
Fungi Diaporthales Gnomoniaceae Gnomoniella	
Fungi Phyllachorales Phyllachoraceae Coccodiella chamaedoraeae	
Animalia Hemiptera	
Animalia Lepidoptera Crambidae Eoophyla	
Animalia Coleoptera Brentidae Trachelizus	
Animalia Hemiptera Oxycarenidae Oxycarenus luctuosus	
Animalia Hemiptera Membracidae Umbonia spinosa	
Animalia Hemiptera Cicadellidae Goniagnathus	
Animalia Hemiptera Liviidae	
Animalia Thysanoptera Thripidae Thrips pusillus	
Animalia Orthoptera Gryllidae Hapithus	NO
Animalia Coleoptera Curculionidae Tomolips	
Animalia Lepidoptera	NO

Insect Species Name	Classification Result (NO: Non-pests)
Animalia Hymenoptera Cynipidae Neuroterus sculpturatus	
Animalia Hemiptera Aleyrodidae Aleurotrachelus dryandrae	
Fungi Peronosporales Peronosporaceae Peronospora trigonellae	
Animalia Hemiptera Aphrophoridae Philaenus	
Animalia Coleoptera Chrysomelidae Bruchus rufipes	
Animalia Hemiptera Aleyrodidae Aleurocanthus t-signatus	
Animalia Hemiptera Rhopalidae Rhopalus parumpunctatus	
Fungi Xylariales Xylariaceae Biscogniauxia rosacearum	
Animalia Isoptera Kalotermitidae	
Fungi Hypocreales Nectriaceae Calonectria	
Animalia Hemiptera Diaspididae Pinnaspis theae	
Fungi Rhytismatales Rhytismataceae Lophodermium	
Animalia Hemiptera Diaspididae Velataspis	
Animalia Diptera Tephritidae Paroxyna	
Animalia Hemiptera Pentatomidae Carbula	
Animalia Thysanoptera Thripidae Psydrothrips kewi	
Animalia Hemiptera Coccidae Saissetia	
Animalia Hemiptera Oxycarenidae Oxycarenus pallens	
Animalia Hemiptera Diaspididae Aspidiotus rigidus	
Animalia Trombidiformes Eriophyidae Aceria zelkoviana	
Animalia Isoptera	
Animalia Coleoptera Curculionidae Catolethrus longulus	
Animalia Coleoptera Scarabaeidae Stenocrates	
Animalia Coleoptera Elateridae Conoderus tumidicollis	
Animalia Hemiptera Scutelleridae Calidea	

**Insect Species Name**	**Classification Result (NO: Non-pests)**
Fungi Phyllachorales Phyllachoraceae Coccostromopsis chamaedoreae	
Animalia Coleoptera Apionidae Chrysapion	
Fungi Not Assigned Not Assigned Chalara urceolata	
Animalia Coleoptera Curculionidae Blosyrus asellus	
Animalia Hemiptera Cicadellidae	
Animalia Coleoptera Chrysomelidae Lithraeus pyrrhomelas	
Animalia Coleoptera Apionidae Protapion nigritarse	
Animalia Hemiptera Aleyrodidae Aleuroclava hikosanensis	
Animalia Coleoptera Buprestidae Agrilus planipennis	
Plantae Fabales Fabaceae Prosopis articulata	NO
Animalia Hemiptera Diaspididae Diaspidiotus	
Animalia Hemiptera Pseudococcidae Ferrisia	
Animalia Hymenoptera Eulophidae	
Animalia Hemiptera Membracidae Ochropepla pallens	
Animalia Hemiptera Pentatomidae Agonoscelis haraldi	
Animalia Coleoptera Bostrichidae Trogoxylon spinifrons	
Animalia Hemiptera Coreidae Hypselonotus	
Animalia Lepidoptera Pyralidae Mussidia	
Animalia Lepidoptera	NO
Animalia Lepidoptera Noctuidae	NO
Animalia Orthoptera Tetrigidae Paratettix	NO
Animalia Coleoptera Buprestidae Acmaeodera vincina	
Animalia Hemiptera Pseudococcidae Pseudococcus	
Animalia Hemiptera Aleyrodidae Aleuroclava euphoriae	
Animalia Hemiptera Rhyparochromidae Valtissius	

Insect Species Name	Classification Result (NO: Non-pests)
Animalia Coleoptera Curculionidae Crypturgus	
Animalia Lepidoptera Choreutidae	NO
Animalia Orthoptera Gryllacrididae	NO
Animalia Coleoptera Curculionidae Cholus viduatus	
Animalia Hemiptera Pseudococcidae Maconellicoccus multipori	
Fungi Capnodiales Mycosphaerellaceae Cercosporidium	
Animalia Hemiptera Miridae Calocoris	
Fungi Glomerellales Glomerellaceae Colletotrichum cordylinicola	
Animalia Coleoptera Scarabaeidae Protaetia	
Animalia Orthoptera Acrididae Brachystola magna	NO
Animalia Lepidoptera Momphidae	NO
Animalia Hemiptera Cicadellidae Plesiommata	
Plantae Alismatales Hydrocharitaceae Hydrilla	NO
Animalia Hemiptera Pseudococcidae Formicococcus polysperes	
Plantae Poales Poaceae Leptochloa	NO
Fungi Capnodiales Davidiellaceae Cladosporium arthrinioides	
Animalia Coleoptera Cerambycidae Chlorophorus signaticollis	
Animalia Lepidoptera Geometridae Gelasma	NO
Fungi Diaporthales Diaporthaceae Phomopsis cocoina	
Fungi Capnodiales Mycosphaerellaceae Cercospora menthicola	
Animalia Hemiptera Coccidae Lichtensia	
Animalia Coleoptera Cerambycidae Coleoxestia	
Animalia Hemiptera Coreidae Centrocoris	
Animalia Coleoptera Coccinellidae	NO
Animalia Lepidoptera Tineidae Opogona sacchari	

Insect Species Name	Classification Result (NO: Non-pests)
Animalia Trombidiformes Tenuipalpidae Brevipalpus	
Animalia Hemiptera Pentatomidae Loxa	
Animalia Hemiptera Coccidae Ceroplastes rusci	
Animalia Hemiptera Cicadellidae Agrosoma proxima	
Fungi Ustilaginales	
Animalia Hemiptera Pentatomidae Scotinophara	
Animalia Coleoptera Chrysomelidae Charidotis vitreata	
Animalia Lepidoptera Geometridae Pantherodes colubraria	NO
Fungi Pleosporales Not Assigned Ascochyta pterophila	
Animalia Hemiptera Aphididae Eulachnus	
Animalia Hemiptera Pseudococcidae Hypogeococcus othnius	
Animalia Coleoptera Curculionidae Scolytogenes	
Animalia Hemiptera Tingidae Atheas	
Animalia Hemiptera Tingidae Acalypta	
Fungi Ustilaginales Glomosporiaceae Glomosporium	
Animalia Lepidoptera Erebidae Letis	NO
Fungi Pleosporales Didymellaceae Didymella	
Fungi Rhytismatales Rhytismataceae Rhytisma	
Animalia Trombidiformes Eriophyidae Acalitus phloeocoptes	
Fungi Pucciniales Pucciniaceae Puccinia petroselini	
Animalia Diptera Cecidomyiidae Campylomyza	
Animalia Hemiptera Aleyrodidae Aleuroclava psidii	
Animalia Lepidoptera Crambidae Neoleucinodes elegantalis	
Animalia Hemiptera Rhopalidae Niesthrea	
Animalia Hemiptera Diaspididae Diaspidiotus alni	

Insect Species Name	Classification Result (NO: Non-pests)
Animalia Hemiptera Aleyrodidae Paraleyrodes	
Fungi Capnodiales Mycosphaerellaceae Septoria centellae	
Animalia Lepidoptera Tortricidae Ecdytolopha	
Animalia Hemiptera Tingidae Lasiacantha hedenborgii	
Animalia Coleoptera Cerambycidae Ergates	
Animalia Hemiptera Pseudococcidae Exallochlus philippinensis	
Animalia Hemiptera Aleyrodidae Aleurodicus capiangae	
Animalia Hemiptera Diaspididae Diaspidiotus maleti	
Fungi Capnodiales Mycosphaerellaceae Cercospora echinodori	
Animalia Stylommatophora Limacidae Limacus	NO
Animalia Coleoptera Cerambycidae Oxymerus luteus	
Animalia Hemiptera Rhyparochromidae Pamerarma ventralis	
Animalia Orthoptera Mogoplistidae Cycloptilum	NO
Animalia Hemiptera Pachygronthidae Oedancala mexicana	
Animalia Hemiptera Oxycarenidae Crophius	
Animalia Hemiptera Miridae Apachemiris vigilax	
Animalia Hemiptera Pseudococcidae Dysmicoccus caribensis	
Animalia Lepidoptera Erebidae Eulepidotis guttata	NO
Animalia Lepidoptera Sphingidae Xylophanes	NO
Animalia Hemiptera Miridae Callichila picticollis	
Animalia Coleoptera Curculionidae Pantomorus albicans	
Animalia Thysanoptera Thripidae Synaptothrips gezinae	
Animalia Hemiptera Diaspididae Diaspis gilloglyi	
Animalia Hemiptera Cicadellidae Xestocephalus	
Animalia Dorylaimida Longidoridae Xiphinema simile	

**Insect Species Name**	**Classification Result (NO: Non-pests)**
Animalia Coleoptera Curculionidae Cholus	
Animalia Hemiptera Delphacidae	
Animalia Trombidiformes Tarsonemidae Deleonia	
Animalia Hemiptera Rhyparochromidae Panaorus	
Animalia Tylenchida Pratylenchidae Pratylenchus delattrei	
Animalia Coleoptera Curculionidae Hylobius	
Animalia Coleoptera Curculionidae Sibinia inornata	
Fungi Pucciniales Not Assigned Uredo musae	
Animalia Hemiptera Miridae Campylomma incerta	
Animalia Orthoptera Gryllidae Gryllus campestris	NO
Animalia Diptera Tephritidae	
Animalia Hemiptera Aetalionidae Aetalion gratum	
Plantae Asterales Asteraceae Onopordum acaulon	NO
Animalia Coleoptera Cerambycidae Chlorophorus strobilicola	
Animalia Hemiptera Rhyparochromidae Remaudiereana annulipes	
Animalia Coleoptera Cerambycidae Eburia fisheri	
Animalia Coleoptera Chrysomelidae Bruchus dentipes	
Animalia Coleoptera Chrysomelidae Symbrotica mexicana	
Fungi Pucciniales Melampsoraceae	
Animalia Hemiptera Alydidae Alydus	
Animalia Hymenoptera Apidae	NO
Animalia Lepidoptera Noctuidae Spragueia	
Animalia Orthoptera Gryllidae Pteronemobius	NO
Animalia Coleoptera Scarabaeidae Ceraspis	
Animalia Hemiptera Pseudococcidae Dysmicoccus lepelleyi	

Insect Species Name	Classification Result (NO: Non-pests)
Animalia Diptera Psilidae	
Animalia Hemiptera Diaspididae Lindingaspis tingi	
Animalia Lepidoptera Geometridae Pantherodes	NO
Animalia Trombidiformes Tenuipalpidae Cenopalpus	
Animalia Hemiptera Pseudococcidae Delottococcus proteae	
Fungi Hypocreales Nectriaceae Fusarium	
Animalia Hemiptera Pentatomidae Acledra	
Animalia Hemiptera Pentatomidae Ochlerus	
Animalia Thysanoptera Thripidae Chaetanaphothrips	
Animalia Lepidoptera Carposinidae Meridarchis scyrodes	
Animalia Coleoptera Chrysomelidae Altica	
Animalia Hemiptera Miridae Orthops	
Animalia Coleoptera Bostrichidae Bostrychoplites	
Animalia Coleoptera Curculionidae Phelypera	
Animalia Hemiptera Miridae Eccritotarsus	
Animalia Coleoptera Curculionidae Eudiagogus	
Fungi Glomerellales Glomerellaceae Colletotrichum scovillei	
Animalia Lepidoptera Erebidae Antichloris viridis	NO
Animalia Hymenoptera Tenthredinidae Monophadnoides	
Viruses Not Assigned Luteoviridae Polerovirus cotton leafroll dwarf virus (CLRDV)	
Animalia Trombidiformes Tetranychidae Oligonychus gossypii	
Animalia Hymenoptera Vespidae Vespula vulgaris	
Animalia Hemiptera Rhyparochromidae Peritrechus	
Animalia Thysanoptera Phlaeothripidae Gynaikothrips	
Chromista Peronosporales Peronosporaceae Phytophthora sojae	

Insect Species Name	Classification Result (NO: Non-pests)
Animalia Hemiptera Diaspididae Oceanaspidiotus pangoensis	
Animalia Hemiptera Diaspididae Melanaspis	
Animalia Lepidoptera Megalopygidae Norape	NO
Animalia Trombidiformes Tarsonemidae Ototarsonemus	
Animalia Coleoptera Elateridae Heteroderes	
Animalia Thysanoptera Aeolothripidae	
Animalia Hemiptera Dinidoridae Megymenum brevicorne	
Animalia Coleoptera Curculionidae Hylastes ater	

Table 7. ChatGPT Classification Results for Additional U.S. Regulated Pest Species from Pest Tracker

Insect Species Scientific Name	Insect Species Common Name	Classification Result (NO: Non-pests)
Alfalfa snout beetle	Otiorhynchus ligustici	
Alfalfa weevil	Hypera postica	
Allium leaf miner	Phytomyza gymnostoma	
Ambrosia beetle	Megaplatypus mutatus	
Ambrosia beetle	Xyleborus spp.	
American palm cixiid	Haplaxius crudus	
Apple ermine moth	Yponomeuta malinellus	
Apple maggot	Rhagoletis pomonella	
Apple Tortrix	Archips fuscocupreanus	
Argentine ant	Linepithema humile	
Arrowhead scale	Unaspis yanonensis	NO
Asian corn borer	Ostrinia furnacalis	
Asian longhorned beetle	Anoplophora glabripennis	
Asian tiger mosquito	Aedes albopictus	

Insect Species Scientific Name	Insect Species Common Name	Classification Result (NO: Non-pests)
Asiatic citrus psyllid	Diaphorina citri	
Asiatic garden beetle	Maladera castanea	
Asiatic rice borer	Chilo suppressalis	
Avocado seed moth	Stenoma catenifer	
Bagrada bug	Bagrada hilaris	
Bamboo borer	Chlorophorus annularis	
Banana moth	Opogona sacchari	
Banded elm bark beetle	Scolytus schevyrewi	
Bark beetle	Pityoborus comatus	
Bark beetle; Engraver beetle	Ips subelongatus	
Bean plataspid	Megacopta cribraria	
Black cutworm	Agrotis ipsilon	
Black fir sawyer	Monochamus urussovii	
Black imported fire ant (IFA)	Solenopsis richteri	
Black maize beetle	Heteronychus arator	NO
Black Parlatoria scale	Parlatoria ziziphi	
Black spruce beetle	Tetropium castaneum	
Black-arched tussock moth	Lymantria monacha	
Blueberry maggot	Rhagoletis mendax	
Boll weevil	Anthonomus grandis	
Box tree moth	Cydalima perspectalis	
Bronze birch borer	Agrilus anxius	
Brown citrus aphid	Toxoptera citricidus	
Brown fir longhorned beetle	Callidiellum villosulum	
Brown marmorated stink bug	Halyomorpha halys	

**Insect Species Scientific Name**	**Insect Species Common Name**	**Classification Result (NO: Non-pests)**
Brown spruce longhorned beetle	Tetropium fuscum	
Bruce spanworm	Operophtera bruceata	
Bud borer	Crocidosema aporema	
Cabbage cluster caterpillar	Crocidolomia binotalis	
Cabbage moth	Mamestra brassicae	
Cactus moth	Cactoblastis cactorum	
Camphor shot borer	Cnestus mutilatus	
Caribbean fruit fly; Carib fly	Anastrepha suspensa	
Carpenter worm	Chilecomadia valdiviana	
Casuarina tussock moth	Lymantria xylina	
Cerambycid beetle	Xylotrechus spp.	
Cerambycid sawyer beetle	Monochamus spp.	
Cereal leaf beetle	Oulema melanopus	
Cereal leaf beetle fairyfly	Anaphes flavipes	
Cherry bark Tortrix	Enarmonia formosana	
Cherry blossom moth	Argyresthia pruniella	
Chilli thrips; Yellow tea thrips	Scirtothrips dorsalis	
Christmas berry webworm	Cryptoblabes gnidiella	
Citrus fruit borer	Gymnandrosoma aurantianum	
Citrus longhorned beetle	Anoplophora chinensis	
City longhorned beetle	Aeolesthes sarta	NO
Cocoa mealybug	Exallomochlus hispidus	
Coconut rhinoceros beetle	Oryctes rhinoceros	
Codling moth	Cydia pomonella	
Colorado potato beetle	Leptinotarsa decemlineata	

Insect Species Scientific Name	Insect Species Common Name	Classification Result (NO: Non-pests)
Coneworms; Dioryctria moths	Dioryctria spp.	
Cotton cutworm	Spodoptera litura	
Cotton seed bug	Oxycarenus hyalinipennis	
Cottony citrus scale	Pulvinaria polygonata	
Cottony cushion scale	Icerya purchasi	
Cucurbit beetle	Diabrotica speciosa	
Diaprepes root weevil	Diaprepes abbreviatus	
Egyptian cottonworm	Spodoptera littoralis	
Elm seed bug	Arocatus melanocephalus	
Elongate hemlock scale	Fiorinia externa	
Emerald ash borer	Agrilus planipennis	
Erythrina gall wasp	Quadrastichus erythrinae	
Euphorbia pit scale	Planchonia stentae	
European chafer	Amphimallon majalis	
European cherry fruit fly	Rhagoletis cerasi	
European chestnut weevil	Curculio elephas	
European corn borer egg parasite	Trichogramma ostriniae	
European crane fly	Tipula paludosa	
European elm bark beetle	Scolytus scolytus	
European elm flea weevil	Orchestes alni	
European grape berry moth	Eupoecilia ambiguella	
European grapevine moth	Lobesia botrana	
European hardwood ambrosia beetle	Trypodendron domesticum	
European honey bee	Apis mellifera european	NO
European oak bark beetle	Scolytus intricatus	

Insect Species Scientific Name	Insect Species Common Name	Classification Result (NO: Non-pests)
European oak borer	Agrilus sulcicollis	
European pepper moth	Duponchelia fovealis	
European pine sawfly	Neodiprion sertifer	
European pine shoot moth	Rhyacionia buoliana	
European poplar shoot borer	Gypsonoma aceriana	
European Shothole Borer	Anisandrus dispar	
European spruce bark beetle	Ips typographus	
European spruce beetle	Dendroctonus micans	
Fall webworm	Hyphantria cunea	
False coconut scale	Aspidiotus rigidus	
False codling moth	Thaumatotibia leucotreta	
Flighted spongy moth complex	Lymantria dispar asiatica	
Flighted spongy moth complex	Lymantria umbrosa	
Flighted spongy moth complex	Lymantria postalba	
Flighted spongy moth complex	Lymantria albescens	
Flighted spongy moth complex	Lymantria dispar japonica	
Forest tent caterpillar	Malacosoma disstria	
Formosan subterranean termite	Coptotermes formosanus	
Fruit piercing moth	Eudocima fullonia	
Fruit tree Tortrix	Archips podana	
Giant wood wasp	Urocerus gigas gigas	
Glassy-winged sharpshooter	Homalodisca vitripennis	
Golden twin spot moth	Chrysodeixis chalcites	
Goldspotted oak borer	Agrilus auroguttatus	
Granulate Ambrosia beetle	Xylosandrus crassiusculus	

Insect Species Scientific Name	Insect Species Common Name	Classification Result (NO: Non-pests)
Green alder sawfly	Monsoma pulveratum	NO
Green oak Tortrix	Tortrix viridana	
Guatemalan potato moth	Tecia solanivora	
Hemlock borer	Melanophila fulvoguttata	
Hemlock looper	Lambdina fiscellaria	
Hemlock woolly adelgid	Adelges tsugae	
Hemlock woolly adelgid lady beetle	Sasajiscymnus tsugae	
Hibiscus (Pink) mealybug	Maconellicoccus hirsutus	
Hickory shuckworm	Cydia caryana	
Horned oak gall wasp	Callirhytis cornigera	
Horse chestnut leafminer	Cameraria ohridella	
Iceryine scale	Crypticerya multicicatrices	
Iris borer	Macronoctua onusta	
Japanese beetle	Popillia japonica	
Japanese cedar longhorned beetle	Callidiellum rufipenne	
Japanese pine sawyer	Monochamus alternatus	
Japanese wax scale	Ceroplastes japonicus	
Khapra beetle	Trogoderma granarium	
Ladybird beetle	Stethorus punctum	NO
Ladybird beetle scale predator	Chilocorus kuwanae	
Large pine weevil	Hylobius abietis	
Large white butterfly	Pieris brassicae	
Leek moth	Acrolepiopsis assectella	
Lesser pine shoot beetle	Tomicus minor	
Lesser spruce shoot beetle	Hylurgops palliatus	

Insect Species Scientific Name	Insect Species Common Name	Classification Result (NO: Non-pests)
Light brown apple moth	Epiphyas postvittana	
Lily leaf beetle	Lilioceris lilii	
Lime swallowtail	Papilio demoleus	
Little fire ant	Wasmannia auropunctata	
Lobate lac scale	Paratachardina pseudolobata	
Locust leafminer	Odontota dorsalis	
Loosestrife root weevil	Hylobius transversovittatus	
Malaysian fruit fly	Bactrocera latifrons	
Mango seed weevil	Sternochetus mangiferae	
Masson pine moth	Dendrolimus punctatus	
Mealybug	Hypogeococcus pungens	
Mediterranean fruit fly (Medfly)	Ceratitis capitata	
Mediterranean pine engraver	Orthotomicus erosus	
Melon fly	Bactrocera cucurbitae	
Mexican bean beetle	Epilachna varivestis	
Mexican fruit fly (Mexfly)	Anastrepha ludens	
Mexican rice borer	Eoreuma loftini	
Mormon cricket	Anabrus simplex	
Mottled umber moth	Erannis defoliaria	
Mountain oak longhorned beetle	Massicus raddei	
Mountain pine beetle	Dendroctonus ponderosae	
Mulberry borer	Xylotrechus chinensis	
Multicolored Asian lady beetle	Harmonia axyridis	
Nettle caterpillar	Darna pallivitta	
New guinea sugarcane weevil	Rhabdoscelus obscurus	

Insect Species Scientific Name	Insect Species Common Name	Classification Result (NO: Non-pests)
Noctuid moth	Copitarsia spp.	
Oak Ambrosia beetle	Platypus quercivorus	
Oak processionary moth	Thaumetopoea processionea	
Oak splendour beetle	Agrilus biguttatus	
Old World bollworm	Helicoverpa armigera	
Olive fruit fly	Bactrocera oleae	
Orange spiny whitefly	Aleurocanthus spiniferus	
Oriental fruit fly	Bactrocera dorsalis	
Oriental fruit moth	Grapholita molesta	
Palm borer	Paysandisia archon	
Passionvine mealybug	Planococcus minor	
Peach fruit fly	Bactrocera zonata	
Pear leaf blister moth	Leucoptera malifoliella	
Pear thrips	Taeniothrips inconsequens	
Pecan nut casebearer	Acrobasis nuxvorella	
Pecan weevil	Curculio caryae	
Pine beauty moth	Panolis flammea	
Pine processionary moth	Thaumetopoea pityocampa	
Pine sawfly	Diprion pini	
Pine shoot beetle	Tomicus piniperda	
Pine shoot beetle	Tomicus destruens	
Pine tortoise scale	Toumeyella parvicornis	
Pine-tree lappet	Dendrolimus pini	
Pink bollworm	Pectinophora gossypiella	
Plum Curculio	Conotrachelus nenuphar	

Insect Species Scientific Name	Insect Species Common Name	Classification Result (NO: Non-pests)
Plum fruit moth	Grapholita funebrana	
Poplar borer	Saperda calcarata	
Potato psyllid	Bactericera cockerelli	
Potato Tuberworm	Phthorimaea operculella	
Red imported fire ant	Solenopsis invicta	
Red palm weevil	Rhynchophorus ferrugineus	
Redbay Ambrosia beetle	Xyleborus glabratus	
Redhaired pine bark beetle	Hylurgus ligniperda	
Rice stink bug	Tibraca limbativentris	
Rosy tussock moth	Lymantria mathura	
Rough sweetpotato weevil	Blosyrus asellus	
Russian wheat aphid	Diuraphis noxia	
Rustic borer	Xylotrechus colonus	
Sakhalin pine sawyer	Monochamus saltuarius	
Sakhalin silk moth	Dendrolimus superans	
Satin moth	Leucoma salicis	
Scarlet oak sawfly	Caliroa quercuscoccineae	
Siberian silk moth	Dendrolimus sibiricus	
Silver Y moth	Autographa gamma	
Sirex woodwasp	Sirex noctilio	
Sixspined Ips	Ips calligraphus	
Sixtoothed bark beetle	Ips sexdentatus	
Sixtoothed spruce bark beetle	Pityogenes chalcographus	
Slender-banded pinecone longhorn beetle	Chlorophorus strobilicola	
Small brown planthopper	Laodelphax striatellus	

Insect Species Scientific Name	Insect Species Common Name	Classification Result (NO: Non-pests)
Small hive beetle	Aethina tumida	
Small white-marmorated longhorned beetle	Monochamus sutor	
Soft wax scale	Ceroplastes destructor	
South American palm weevil	Rhynchophorus palmarum	
South American rice miner	Hydrellia wirthi	
Southern mole cricket	Scapteriscus borellii	
Southern pine beetle	Dendroctonus frontalis	
Soybean aphid	Aphis glycines	
Soybean pod borer	Maruca vitrata	
Soybean stem borer	Dectes texanus	
Spongy moth	Lymantria dispar	
Spongy Moth	Lymantria dispar dispar	
Spotted lanternfly	Lycorma delicatula	
Spotted stem borer	Chilo partellus	
Spotted wing Drosophila	Drosophila suzukii	
Spring cankerworm	Paleacrita vernata	
St. johnswort borer	Agrilus hyperici	
Stalk borer	Papaipema nebris	
Strawberry blossom weevil	Anthonomus rubi	
Summer fruit Tortrix moth	Adoxophyes orana	
Sunn pest	Eurygaster integriceps	
Swede midge	Contarinia nasturtii	
Sweetpotato whitefly	Bemisia tabaci	
Tawny crazy ant	Nylanderia fulva	
Tomato fruit borer	Neoleucinodes elegantalis	

Insect Species Scientific Name	Insect Species Common Name	Classification Result (NO: Non-pests)
Tomato leaf miner	Phthorimaea absoluta	
Tremex woodwasp	Tremex fuscicornis	
Tropical Fire ant	Solenopsis geminata	
Tuber flea beetle	Epitrix tuberis	
Tufted apple bud moth	Platynota idaeusalis	
Twobanded Japanese weevil	Pseudocneorhinus bifasciatus	
Twospotted citrus psyllid	Trioza erytreae	
Variegated golden Tortrix	Archips xylosteanus	
Velvet longhorned beetle	Trichoferus campestris	
Viburnum leaf beetle	Pyrrhalta viburni	
Vine mealybug	Planococcus ficus	
Walnut twig beetle	Pityophthorus juglandis	
West indian cane weevil	Metamasius hemipterus	
Western bean cutworm	Striacosta albicosta	
Western corn rootworm	Diabrotica virgifera	
Wheat bug	Nysius huttoni	
White clover weevil	Ischnopterapion virens	
Whitefringed weevil	Naupactus leucoloma	
Whitespotted sawyer	Monochamus scutellatus	
Wingless weevil	Otiorhynchus dieckmanni	
Winter moth	Operophtera brumata	
Yellow peach moth; Castor capsule borer	Conogethes punctiferalis	
Zimmerman pine moth	Dioryctria zimmermani	

Table 8. ChatGPT Classification Results for Common Beneficial Insects in U.S.

Insect Species Name	Classification Result (YES: Pests)
Celer Crab Spider	
Jumping Spider	
Green Lacewings	
Brown Lacewings	
Ground and Tiger Beetles	
Seven-Spotted Lady Beetle	
Asian Lady Beetle	YES
Pink Spotted Lady Beetle	
Collops Beetle	
Hooded Beetle	
Rove Beetle	
Damsel Bugs	
Spined Soldier Bug	
Spined Assassin Bug	
Leafhopper Assassin Bug	
Big-Eyed Bugs	
Syrphid Fly	
Minute Pirate Bug	
Tachinid Fly	
Trichogramma Wasps	

Appendix E. YOLO Model Architecture and Specifications

A CNN-based YOLO model was used as a conventional YOLO-based approach for comparison, providing empirical grounding for evaluating the behavior of the proposed iNaturalist-based identification system on the same field-test images. A summary of the model specifications and computational characteristics is presented in Table 9, while a simplified representation of its CNN architecture is illustrated in Fig. 15..

Table 9. Summary of the YOLO Model Characteristics

Model Characteristic	Value	Description
Input resolution	416 x 416 x 3	RGB image input
Convolutional layers	9	Feature extraction and prediction
Max-pooling layers	6	Spatial downsampling
Leaky ReLU layers	8	Nonlinear activation
Trainable parameters	11.063 M	Learned model parameters
Model size	21.11 MB	TFList model file size
Output shape	13 x 13 x 100	Raw YOLO detection tensor
Output values	16900	Total values in the output tensor
TFList operations	50	Operations in the converted computational graph
Average inference time	52.26 ms/image	Average inference time on the test computer
Approximate FPS	19.13	Approximate images processed per second
Depthwise convolution	No	Standard convolutional layers used
Integer quantization	No	Not INT8/UINT8 quantized

Input

416 × 416 × 3

↓

Conv 3 × 3: 3 → 16

↓ MaxPool

Conv 3 × 3: 16 → 32

↓ MaxPool

Conv 3 × 3: 32 → 64

↓ MaxPool

Conv 3 × 3: 64 → 128

↓ MaxPool

Conv 3 × 3: 128 → 256

↓ MaxPool

Conv 3 × 3: 256 → 512

↓ MaxPool

Conv 3 × 3: 512 → 1024

↓

Conv 3 × 3: 1024 → 512

↓

Conv 1 × 1: 512 → 100

↓

Raw Detection Output

13 × 13 × 100

↓

5 boxes × (4 box coordinates + 1 objectness + **15 classes**)

Fig. 15. Architecture of the CNN-based YOLO insect detection model.
